# Functional specialization for language processing in inferior frontal regions during early childhood: evidence from functional near-infrared spectroscopy individual functional channels of interest approach

**DOI:** 10.1117/1.NPh.12.3.035012

**Published:** 2025-09-13

**Authors:** Haolun Luo, Tao Yu, Qun Li, Li Sheng

**Affiliations:** a The Hong Kong Polytechnic University, Department of Language Science and Technology, Faculty of Humanities, Hong Kong, China; b Shanghai Jiao Tong University, Department of Developmental and Behavioral Pediatrics, Shanghai Children’s Medical Center, Shanghai, China; c West China Hospital of Sichuan University, Department of Otorhinolaryngology-Head and Neck Surgery, Chengdu, China

**Keywords:** cognitive load, development, functional near-infrared spectroscopy, language, multiple demands

## Abstract

**Significance:**

Early language acquisition represents a fundamental achievement in cognitive development, yet the neural mechanisms underlying this process remain debated, particularly whether specialized language regions exist from early life or emerge gradually through development.

**Aim:**

We aim to investigate the functional specialization for language processing in early childhood. We first aimed to validate an individual functional channel of interest (fCOI) approach for dissociating language and cognitive control regions in adults and then to apply this method to examine whether these functional profiles are present in toddlers.

**Approach:**

Using functional near-infrared spectroscopy with the fCOI approach, we conducted two experiments involving adults (N=20, ages 18 to 26 years) and toddlers (N=22, ages 2 to 4 years) who completed language processing (intact versus degraded speech) and cognitive control tasks (spatial working memory task for adults, go/no-go task for toddlers).

**Results:**

For language regions within the left inferior frontal gyrus (LIFG), both adults and toddlers showed a significantly stronger response to intact versus degraded speech, with no significant modulation by cognitive demand manipulation. However, language selectivity in the homologous right hemisphere region was present only in adults. The multiple demand regions showed complementary patterns, with selectivity for cognitive control of regions within the right inferior frontal gyrus (RIFG) emerging early.

**Conclusions:**

These findings provide evidence for early neural specialization of language processing in LIFG while revealing ongoing development in RIFG organization. Our results support models of early language-specific neural regions rather than gradual differentiation from domain-general mechanisms while highlighting the protracted development of language organization.

## 1 Introduction

Language acquisition in early childhood represents a remarkable achievement of human cognitive development, occurring naturally through exposure without formal training.^1^ A fundamental question in developmental cognitive neuroscience is how the brain supports early language acquisition. One of the most debated aspects of language development is whether the brain contains specialized circuits for language processing from early in life, or whether these circuits emerge gradually over development. Specifically, there are two competing hypotheses about the neural mechanisms underlying language processing in young children. One possibility is that language processing relies on domain-specific neural mechanisms from early on, suggesting an early neural specialization for language that is distinct from other cognitive functions.^2^^,^^3^ Alternatively, language processing might initially emerge through domain-general cognitive processes, such as working memory and executive function, with specialized language circuits developing gradually through experience.^4^^,^^5^

Studies in adults, primarily using functional magnetic resonance imaging (fMRI), have identified two distinct neural networks potentially relevant for understanding language development: the “core language network” and the “multiple demand” (MD) network. The language network, which includes regions primarily in left frontal and temporal areas, shows consistent engagement during language processing with limited activation during nonlinguistic tasks.^6^^–^^8^ By contrast, the MD network, comprising bilateral frontal, parietal, cingulate, and insular regions, responds to increased cognitive demands across various cognitive tasks.^9^^,^^10^ Although some researchers have argued that language processing relies substantially on the domain-general mechanisms of the MD network,^11^^,^^12^ recent adult neuroimaging evidence supports more distinct functional roles.^13^ These studies suggested that the language network implements core computations for linguistic structure and meaning, whereas MD areas are recruited when task requirements demand increased cognitive control.^13^^–^^25^ This evidence suggests a functional dissociation in the mature brain.

Although studies with adults have suggested a relatively clear dissociation between language-specific and domain-general networks, documenting this dissociation in adults alone is insufficient for understanding the origin of unique human cognitive abilities. Several competing developmental trajectories could lead to the same adult organization: cortical regions might start as domain-general and gradually specialize through competitive interactions,^4^ with language regions initially participating in broader cognitive functions such as working memory before gaining linguistic selectivity. Alternatively, domain-general processes might bootstrap the development of language abilities.^26^^–^^28^ A third pattern could be that there is an innate proto-organization of domain-specificity. Understanding how and when these networks dissociate during development is therefore critical for distinguishing between these possibilities and illuminating the origins of human cognitive architecture. Recent fMRI studies have demonstrated that this selective and specific language network is already established in children as young as 4 years of age, showing adult-like functional dissociation from domain-general cognitive processes.^29^^,^^30^ However, due to the methodological constraints of fMRI, particularly the requirement for participants to remain still, the emergence and organization of these networks in even younger children remains largely unexplored. To the best of our knowledge, there is no existing exploration of the double dissociation of language and domain-general cortex in children younger than 4.

To address this gap, we employ functional near-infrared spectroscopy (fNIRS), which allows measurements while participants move relatively freely. fNIRS uses near-infrared light emitters and detectors placed against the scalp to estimate changes in oxygenated and deoxygenated hemoglobin concentrations, which are derived from how the properties of light are altered by tissue absorption and scattering.^31^ This method has been successfully used to study early language processing in infants. Several studies have demonstrated that newborns already show specialized neural responses to speech in temporal and frontal regions,^32^^–^^34^ whereas later work has suggested that language-specific responses continue to develop throughout infancy.^35^ Although these studies provided evidence for differential neural responses to speech versus reversed or degraded speech stimuli, they have not fully characterized the functional architecture of language processing in the developing brain. Specifically, showing greater activation to speech than nonspeech does not establish whether these regions are specialized for language processing or serve more domain-general functions that happen to be engaged during speech processing. As Kanwisher^36^ emphasizes, in studies of functional specificity, demonstrating true specialization not only requires evidence of preferential responses to one category but should also test whether regions are exclusively engaged in processing that category. For example, the right temporoparietal junction activates selectively when thinking about others’ thoughts, but not during closely related tasks such as processing physical appearances or even other mental states such as hunger or pleasure.^37^ In addition to these conceptual challenges, mapping fNIRS measurement channels to specific cortical regions presents several methodological challenges, particularly in developmental populations. These challenges arise from anatomical variability in the relationship between external landmarks and underlying cortical structures, developmental variations in head size affecting measurement array coverage, and practical constraints in precise optode placement. Moreover, even when measurement channels can be accurately mapped to specific anatomical regions, this anatomical localization does not guarantee functional correspondence. A given anatomical region often contains multiple functionally distinct subregions that vary in size and precise location across individuals.^38^^,^^39^

To overcome these methodological challenges, we employ a functional channel of interest (fCOI) approach for analyzing fNIRS data.^40^ Unlike traditional approaches, which assume that measurement channels correspond to the same cortical regions across participants, the fCOI method identifies channels of interest by examining each individual’s functional activation patterns. The fCOI approach offers several key advantages for developmental studies^41^: it accounts for individual variability in brain anatomy and functional organization,^42^ reduces noise from neighboring regions with different functional profiles, and provides a principled way to limit multiple comparisons while maintaining statistical power. Indeed, its utility has been demonstrated in fNIRS studies of infant and young children across multiple domains of cognitive development, including face processing and theory of mind.^40^^,^^43^^,^^44^

In the present study, we investigate the functional organization of language and domain-general networks in toddlers aged 2 to 4 years using fNIRS with the fCOI approach. The toddler period is uniquely suited for this investigation as it represents a critical convergence of cognitive and linguistic maturation.^45^^,^^46^ It is during this window that children first develop the capacity for structured cognitive control necessary to perform tasks such as the Go/No-go paradigm, allowing for a valid assessment of the MD regions. We focus on bilateral IFG regions given their well-documented heterogeneity in adult neuroimaging studies.^13^ Specifically, prior research has established that Broca’s area within the left IFG contains two functionally distinct regions: a language-selective region and a domain-general region.^13^ Although anatomically adjacent, these regions belong to different networks. Moreover, IFG’s anatomical characteristics^47^ (e.g., minimal hair interference, relatively thin skull in this region, proximity to cortical surface) maximize fNIRS signal quality. Therefore, IFG provides an optimal starting point for investigating whether specialized circuits emerge in core regions before extending to broader networks. Our experimental design includes a child-friendly language localizer adapted from Scott et al.,^48^ which contrasts responses to auditorily presented engaging narrative clips versus acoustically degraded versions of the same stimuli. This localizer has been demonstrated to reliably identify language-selective regions across 45 languages from 12 language families, showing consistent activation patterns in the fronto-temporal language network regardless of the specific language being processed.^49^ The passive listening nature of this task makes it particularly suitable for young children who may have difficulty with reading or cognitively demanding tasks, with the confounding effects of task difficulty being controlled. The narrative stimuli can be easily customized with age-appropriate content to maintain children’s attention. For the cognitive control task, we used a spatial working memory task to engage the MD network for adults as this paradigm has been consistently shown to be the most reliable and widely used task for localizing the MD network across numerous studies.^10^^,^^22^ Using the same paradigm allows us to directly compare our fNIRS findings with the extensive fMRI literature. For children, we employed a go/no-go task that probes inhibitory control—another key component of executive function that has been shown to engage the MD network.^50^ Although these tasks tap different aspects of executive function (working memory versus inhibitory control), both have been demonstrated to reliably activate the MD network and show similar patterns of increased activation under higher cognitive demands.^9^^,^^10^ We implemented two conditions in the go/no-go task: an easier condition with only “go” trials and a more demanding condition mixing “go” and “no-go” trials, paralleling the easy versus difficult manipulation in the adult working memory task. This modification was necessary as young children are unable to perform complex spatial working memory tasks^51^ but can engage with the more developmentally appropriate go/no-go paradigm^52^ while still allowing us to probe domain-general cognitive control functions supported by the MD network. Through this approach, we aim to investigate whether these regions show distinct functional profiles for language processing versus domain-general cognitive demands in young children.

To validate our methodology, we first applied the individual fCOI approach to fNIRS data analysis in a sample of adults in experiment 1, ensuring that our source-detector array was capable of detecting activation from known functional regions in the adult cortex. Then, in experiment 2, we examined whether the same language-preferring regions with functional profiles matching those established in the adult literature (i.e., selective responses to linguistic content and insensitivity to cognitive demands) could be observed in toddlers between 2 and 4 years of age using the same approach of identifying fCOIs in individual participants. Specifically, we hypothesized that if functional specialization for language processing is established early in the IFG: (1) In adults, serving as a validation, language fCOIs within the IFG would exhibit selectivity for linguistic content (i.e., greater activation to intact speech versus acoustically degraded speech) and specificity by not responding significantly to manipulations of cognitive demand in a nonlinguistic task. Conversely, MD fCOIs within the IFG would show selectivity for cognitive demand (i.e., greater activation to harder versus easier non-linguistic cognitive control conditions) and specificity by not responding to linguistic content. Evidence of such a double dissociation would validate our approach. (2) In toddlers, if early specialization holds, their language fCOIs in the IFG would similarly show this pattern of selectivity for intact speech and specificity against cognitive demands (from the go/no-go task). Concurrently, their MD fCOIs would be expected to respond to cognitive load but not to the linguistic contrast. Alternatively, if functional specialization emerges more gradually: (1) toddlers’ language fCOIs might show less distinct profiles, potentially responding to both linguistic content and cognitive demands. Findings of overlapping responses or a lack of clear selectivity/specificity in toddlers would contradict a strong early specialization account for the IFG.

## 2 Materials and Methods

### 2.1 Experiment1

#### 2.1.1 Participants

Twenty adults (between 18 and 26 years, 10 female) were recruited from the student population at Sichuan University. All participants had normal or corrected-to-normal vision and no history of neurological or psychiatric disorders, head trauma, seizures, or current use of psychoactive medications. None reported physical impairments that could affect task performance. All were right-handed and gave written informed consent. This study was approved by the Biomedical Ethics Review Committee of West China Hospital, Sichuan University (Protocol #2023-2376) and was conducted in accordance with the Declaration of Helsinki and CIOMS International Ethical Guidelines.

#### 2.1.2 Procedure

**Fig. 1:**
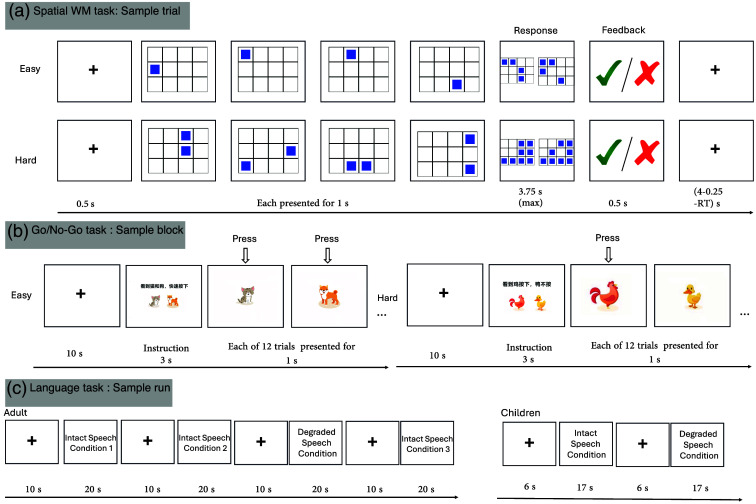
Schematic illustration of the experimental tasks. (a) The spatial working memory (WM) task used for adults (MD localizer). Participants remembered a sequence of blue squares appearing one at a time (easy) or two at a time (hard) in a grid and then selected the correct sequence. (b) The go/no-go task used for toddlers (MD localizer). In the easy condition (go-only), toddlers pressed a button for all stimuli. In the hard condition (go/no-go), they pressed for “go” stimuli (chickens) but withheld their response for “no-go” stimuli (ducks). (c) The language localizer task for adults and toddlers, which involved passive listening to blocks of intact meaningful speech and acoustically degraded speech, with different block timings for each age group.

Participants were seated in front of a 68 cm monitor at a distance of ∼50  cm. Each participant completed (1) an auditory language localizer task^48^ and (2) one spatial working memory task (Fig. 1).^6^

In the language localizer task, participants listened to blocks of intact meaningful sentences and acoustically degraded speech following the procedure introduced by Scott et al.^48^ All the materials for this localizer were adapted to Mandarin from Gweon et al.^37^ and read by a native female speaker. We used female speakers because children tend to pay attention to female voices.^53^ Thirty-two audio clips were created, consisting of 24 intact clips and 8 degraded clips (see the full list of stimuli in the Supplementary Material). Each clip was 19 to 20 s in duration. Degraded speech clips were created from the intact versions using the procedure described below, resulting in muffled speech where the linguistic content was no longer intelligible. The degraded versions were created by first applying a low-pass filter (350 Hz pass-band) to the intact audio clips, then creating a noise track by randomizing the time-points of the original audio, and multiplying it by the amplitude envelope of the intact clip. The noise track was low-pass filtered (8000 Hz pass-band, 10,000 Hz stop frequency) to soften the highest frequencies and added to the filtered speech at a level that rendered it unintelligible. Each block lasted around 20 s. Each run included three blocks corresponding to three language conditions plus one block of the degraded speech condition, with 10-s fixation blocks between blocks and additional fixation blocks at the beginning and end of the run. There are a total of eight runs. The task was originally designed to serve a dual purpose: localizing language processing and theory of mind networks. However, pilot testing revealed that children could not sustain this lengthy protocol, leading us to simplify the design to a single language condition for child participants in experiment 2. For adult participants, we maintained all three language conditions but combined them into a single intact speech condition for analysis, contrasting it with the degraded speech condition. This resulted in an uneven distribution of stimuli between conditions (24 intact versus 8 degraded clips). The presentation order of these intact and degraded speech blocks within each run was systematically varied and counterbalanced across participants to control for potential order effects. Participants were told that they would listen to some fun audio clips and some clips that were distorted in a way that makes it impossible to understand what the speaker is saying. They were instructed to listen attentively. Prior to the experiment, it was ensured that the volume level was sufficiently loud yet comfortable.

In the MD localizer task, participants performed a spatial working memory (WM) task involving a 3×4 grid. During each trial, squares appeared in different locations on the grid, either one at a time (easy condition) or two at a time (hard condition). Participants were required to remember these sequential locations. At the end of each trial, they were shown two sets of locations and had to select the set that matched the sequence they had just seen. They received feedback, indicating whether their response was correct or incorrect.^6^ Each trial followed a precise timing structure: beginning with a 500 ms fixation cross, followed by sequential grid presentations where each location was shown for 1000 ms. Participants then had up to 3750 ms to make their choice between the two location sets. After responding, they received feedback for 250 ms, followed by a fixation cross that filled the remaining time to ensure a consistent trial length (calculated as 4000 ms minus the response time minus 250 ms). Each run consisted of two blocks (one easy, one hard) with four trials per block, plus three 10-s fixation blocks interspersed at the beginning, middle, and end. Participants completed four runs in total. To ensure task comprehension, all participants completed a practice run before beginning the main experiment.

After each run, the display paused until the participant initiated the next run by pressing a key on a keyboard located to his/her right. Participants were instructed to remain still and to focus on the screen throughout each run but were not asked to fixate on any point on the screen and could adjust their position between runs.

#### 2.1.3 Data acquisition

**Fig. 2:**
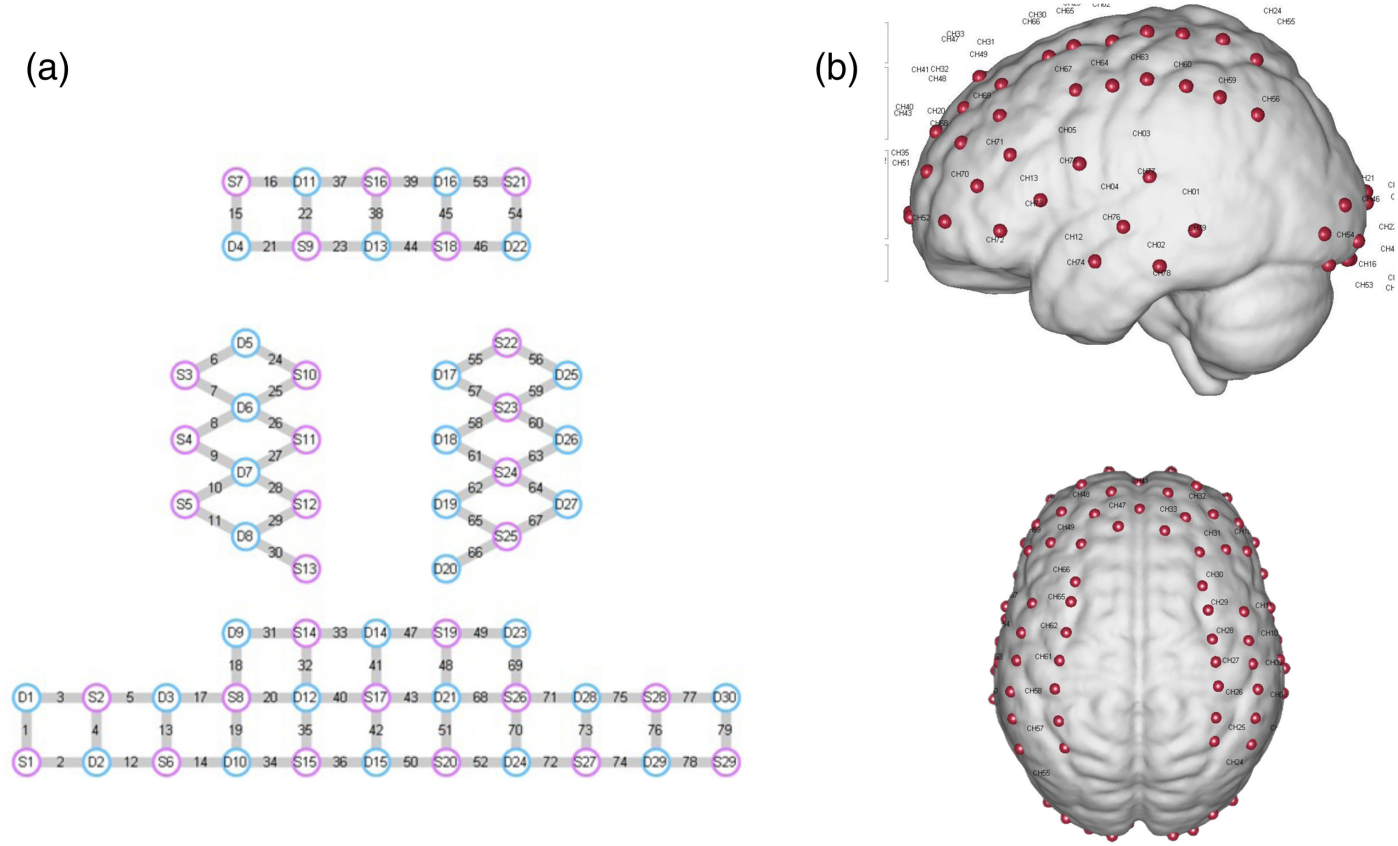
(a) Schematic representation of the fNIRS optode placement. Red circles indicate sources, blue circles indicate detectors, and yellow lines represent measurement channels. (b) Spatial registration result of the probe array. Red dots with numbers indicate channel positions on the MNI standard brain template.

fNIRS measurements were collected using a portable, multichannel continuous-wave NirSmart fNIRS system (Danyang Huichuang Medical Equipment Co., Ltd., China). This system employed LED light sources emitting at wavelengths of 760 and 850 nm, and light attenuation was recorded by avalanche photodiode detectors with a sampling rate of 11 Hz. 29 sources and 30 detectors constituted 79 measurement channels of 3 cm (Fig. 2). The emitter and detector were placed according to the 10 to 20 system. The optodes were stabilized using a plastic holder and then affixed to participants’ heads over the frontal, temporal, occipital, and parietal lobes in the left and right hemispheres using custom headgear. Prior to data collection, head circumference measurements were taken to ensure all participants fell within the suitable range for our standard cap size (54 to 58 cm). When wearing the optode cap, it was ensured that the Cz point of the electrode cap coincided with the Cz point of the scalp surface measurement. The spatial coordinates of sources, detectors, and anchor points (Nz, Cz, Al, Ar, lz, and other points from the international 10 to 20 system) were digitized using an electromagnetic 3D digitizer (Patriot, Polhemus, Colchester, Vermont, United States). Coordinates were then superimposed on a cerebral cortex atlas using the statistical parametric mapping for near-infrared spectroscopy (NIRS-SPM) toolbox.

#### 2.1.4 Data preprocessing

Initial measurements of incident light reaching the detector for each channel were trimmed to remove excess time points from the beginning and end of data collection and were then preprocessed using the HomER NIRS processing package (v1.81.4).^54^ First, each run of the experiment was isolated from the continuous data and processed as a separate file to ensure that normalization of a given run was not skewed by more or less noisy portions of the data occurring at other times in the experiment.^43^ Then, the negative intensity values resulting from noisy data were corrected using the hmrR_PreprocessIntensity_Negative function. Subsequently, channels with a poor signal-to-noise ratio in the raw signal were removed from analyses using the hmrR_PruneChannels function ( dRange = [1e3 1e7] and SNRthresh = 5). This automated pruning was supplemented by visual inspection, and channels still lacking a clear cardiac signal were manually excluded from individual participants’ data sets. Raw intensity measurements were then converted to optical density changes (ΔOD). Then, a linear fit function was used to further remove the baseline drift. Next, a temporal derivative distribution repair (TDDR) was used to filter out motion artifacts.^55^ TDDR was selected for its efficacy in removing common motion-induced artifacts (baseline shifts and spikes), particularly prevalent in data from young children,^55^ without the need for user-supplied parameters. Moreover, TDDR is designed to correct for motion while aiming to preserve the integrity of large, true neural activations, which is vital for accurately characterizing task-related hemodynamic responses. This approach can offer advantages over methods that might require extensive parameter tuning or risk attenuating genuine neural signals in noisy developmental datasets. The resulting time courses were then band-pass filtered with cut-off frequencies of 0.01 to 0.09 Hz to further remove slow drifts and high-frequency and physiological noise^56^ (Pinti et al., butter filter 3 order for lowpass, 5 order for high pass). After that, the ΔOD values were transformed into changes in oxyhemoglobin (HbO2) and deoxyhemoglobin (HHb) concentration according to the modified Beer–Lambert law (hmrOD2Conc, ppf = [1.0 1.0]). Then, to further address systemic physiological noise (e.g., heart rate, respiration, blood pressure changes) that can introduce global, nonneural components into the fNIRS signal, we employed a principal-component-analysis-based spatial filter^57^^,^^58^ (σ=46  deg). This method distinguishes focal neural activity from diffuse systemic interference by identifying widespread signal components common across many channels. A large Gaussian smoothing kernel applied to the PCA spatial patterns ensures that only these global components are regressed out, preserving localized neural signals.^59^ This approach is well-suited for our study, given our extensive coverage, which aids in robustly separating global from focal patterns. Moreover, the concentration of each block is baseline corrected by 2 s prior to the onset of stimuli.

#### 2.1.5 Data analysis

We focused our analysis on changes in oxygenated hemoglobin concentration as it has been demonstrated to be a more sensitive and reliable measure compared with deoxygenated or total hemoglobin concentration changes, which do not provide additional information beyond HbO2.^60^ For each participant and channel, we computed the mean change in HbO2 concentration during each block, beginning 5 s post-stimulus onset to account for hemodynamic response lag. The analysis window extended over the block length (20 s post-onset for the language localizer task and 33 s post-onset for the spatial working memory task), as is commonly done in fNIRS studies.^60^^–^^66^ This block-averaging method was chosen because it provides a direct means of identifying the maximal contrast required for our leave-one-run-out cross-validation procedure. The approach is consistent with prior fNIRS studies that used an individual fCOI methodology^40^^,^^41^^,^^43^^,^^44^ and is further supported by findings that, for block designs, block-averaging and general-linear-model-based analyses can yield comparable group-level experimental conclusions.^60^ The differences in these responses across conditions were then analyzed using the fCOI approach.

First, we selected a set of channels (“search space”)^40^ that plausibly covered regions of the scalp plausibly overlaying bilateral inferior frontal lobe across participants (see Table 1 for probabilistic mapping of selected fNIRS channels to underlying cortical regions).

**Table 1:** Probabilistic mapping of fNIRS channels to underlying cortical regions. The anatomical labeling and corresponding MNI coordinates for each source-detector pair were estimated using the NIRS-SPM software package. Percentages indicate the probability of the channel’s cortical projection falling within the specified Brodmann areas.

Channel	MNI coordinates	Anatomic label	Percentage (%)	Search space
5	64.9643, 5.7288, 27.8566	4—primary motor cortex, 6—pre-motor and supplementary motor cortex, 43—subcentral area, 44—pars opercularis	6.35, 59.53, 27.42, 6.69	RIFG
13	61.2752, 22.4516, 12.4687	6—pre-motor and supplementary motor cortex, 44—pars opercularis, 45—pars triangularis, 48—retrosubicular area	5.75, 31.95, 51.12, 11.18	RIFG
14	57.049, 37.9202, −2.4401	38—temporopolar area, 45—pars triangularis, 46—dorsolateral prefrontal cortex, 47—inferior prefrontal gyrus	3.34, 66.22, 21.40, 9.03	RIFG
17	53.2021, 33.3479, 29.3424	44—pars opercularis, 45—pars triangularis, 46—dorsolateral prefrontal cortex	6.69, 92.57, 0.74	RIFG
19	49.1243, 47.253, 15.4105	45—pars triangularis, 46—dorsolateral prefrontal cortex	48.70, 51.30	RIFG
20	36.6861, 51.5968, 29.9235	9—dorsolateral prefrontal cortex, 45—pars triangularis, 46—dorsolateral prefrontal cortex	4.17, 3.70, 92.13	RIFG
68	−38.2423, 52.8662, 27.4047	45—pars triangularis, 46—dorsolateral prefrontal cortex	12.45, 87.55	LIFG
69	−43.5347, 37.7257, 38.1227	9—dorsolateral prefrontal cortex, 44—pars opercularis, 45—pars triangularis, 46—dorsolateral prefrontal cortex	25.45, 4.55, 39.09, 30.91	LIFG
70	−50.5051, 46.6936, 10.4438	45—pars triangularis, 46—dorsolateral prefrontal cortex	53.45, 46.55	LIFG
71	−54.2983, 33.6329, 22.7694	45—pars triangularis	100	LIFG
72	−56.3003, 37.7076, −7.1488	38—temporopolar area, 45—pars triangularis, 46—dorsolateral prefrontal cortex, 47—inferior prefrontal gyrus	8.14, 48.06, 19.77, 24.03	LIFG
73	−59.7137, 21.7772, 5.0031	6—pre-motor and supplementary motor cortex, 38—temporopolar area, 44—pars opercularis, 45—pars triangularis, 48—retrosubicular area	4.03, 15.44, 14.09, 36.24, 30.20	LIFG
75	−66.014, 6.3358, 19.328	6—pre-motor and supplementary motor cortex, 43—subcentral area, 44—pars opercularis, 48—retrosubicular area	59.79, 31.12, 4.90, 4.20	LIFG

The leave-one-run-out cross-validation procedure^43^ was implemented within each search space, which has demonstrated better sensitivity than other selection methods.^41^ For each participant, one run was held out, whereas the remaining runs were averaged. Within the predefined search space, we identified the language fCOI as the channel showing the maximum contrast between intact speech and degraded speech. This contrast is designed to isolate regions engaged in high-level linguistic processing, such as syntax and semantics, while controlling for the low-level acoustic properties that are matched between the intact and degraded stimuli.^6^^,^^48^ Conversely, the MD fCOI was identified as the channel showing the maximum contrast between the hard and easy conditions of the cognitive control task. This widely used contrast identifies brain regions sensitive to cognitive effort, a core feature of the domain-general multiple-demand network.^10^

Our fCOI selection method does not guarantee the identification of reliably preferential channels in either search space. If none of the available channels recorded from the cortex exhibited distinct responses between conditions, the selection process would just operate on random noise, resulting in no consistent response patterns in the independent data.^40^

To compare the functional profile across the two networks, we also estimated the responses of the fCOIs to conditions that were not used to define them (i.e., hard and easy WM for the language fCOI, intact speech and degraded speech for the MD fCOI). For this analysis, we used all runs from the localizer task to define the fCOIs and all data from the other task to estimate their responses.

For data exclusion, a run was excluded from analysis if more than one-third of the channels within a predefined fCOI search space were rejected due to noise.^44^ A participant would have been excluded from all analyses if they had fewer than two valid runs for any given task. In the present experiment, no participants were excluded based on these criteria.

We employed a generalized linear mixed-effects model on individual trials to account for the unbalanced nature of the dataset (different numbers of runs across participants due to exclusion criteria) and individual differences in global signal strength. The analysis was implemented using MATLAB’s “fitglme” function with the maximum penalized likelihood (MPL) method. Our model structure was: HbO data ∼ condition + (1|subject), where condition represented the experimental conditions (intact versus degraded speech for language task; hard versus easy for cognitive/MD task). Although the inclusion of random slopes for condition by subject would be theoretically appropriate,^67^ attempts to include these led to convergence failures. Separate models were run for two fCOI types in each search space [left inferior frontal gyrus (LIFG) language, right inferior frontal gyrus (RIFG) language, LIFG MD, RIFG MD] of two experiment contrasts (intact > degraded; hard > easy). To account for multiple comparisons, we applied the Bonferroni correction separately for each task (language versus cognitive control). In each task, the left and right IFG fCOIs formed a single family of two comparisons, yielding a family-wise threshold of α=0.025 (0.05/2).

### 2.2 Experiment 2

#### 2.2.1 Participants

Thirty-five children between 2 and 4 years of age were recruited from preschools in the Putuo District of Shanghai, China. Thirteen children were excluded because of failing to follow the experimental instructions. Twenty-two children (mean: 3 years 6 months; range: 2 years 5 months to 4 years 11 months; 14 male, 8 female) were finally included in the study. Two children were aged 2 years 5 months to 2 years 11 months, seventeen were aged 3 years 0 months to 3 years 11 months, and three were aged 4 years 0 months to 4 years 11 months. According to parent reports, all participants had no developmental disorders, had normal or corrected-to-normal vision and hearing, and could understand basic instructions in Mandarin. Although specific data on race/ethnicity were not individually collected, the sample is understood to be predominantly of Han Chinese ethnicity, reflecting the demographics of the recruitment area. Data on parental socioeconomic status, income, or education level were not collected for this study. In addition, all children were full-term births (≥37 weeks gestation) with no significant medical complications at birth. Written informed consent was obtained from parents/legal guardians of all child participants prior to their inclusion in the study, in accordance with the requirements of the Biomedical Ethics Review Committee of West China Hospital, Sichuan University (Protocol #2023-2376). The study was conducted following the principles of the Declaration of Helsinki and CIOMS International Ethical Guidelines

#### 2.2.2 Procedure

The procedure was similar to that of experiment 1. To ensure comfort and compliance, children under 3 years sat on their parent’s lap. Parents were carefully instructed to remain silent and still and to refrain from talking, pointing, or otherwise interacting with their child during the experimental runs. These measures were implemented to ensure that the recorded neural activity was driven solely by the experimental stimuli and not influenced by parent–child interaction. The experiment was discontinued, either during or between runs, if the participant became fussy, inattentive, or if the parent indicated their wish to end the experiment. Each participant completed an auditory language localizer task and a go/no-go task.

In the language localizer task, participants listened to blocks of intact meaningful sentences and acoustically degraded speech following the same procedure described in experiment 1. The materials were adapted from experiment 1 by selecting a subset of the adult stimuli from all three language conditions based on a pre-experiment assessment of comprehension in a separate group of toddlers (n=3). Although the adult version contained three language conditions per run, we selected only the material that showed the highest comprehension rates in our pre-experiment (accurate responses to simple comprehension questions, e.g., “Was the girl helping her father?” or “Was the rabbit in the garden?”). The selected narratives were shortened from the original 20-s duration to 17-s segments to accommodate shorter attention spans while maintaining the core narrative structure. Each block lasted 17 s, with 6-s fixation blocks between blocks and additional fixation blocks at the beginning and end of the run. Each run included one language condition block plus one degraded speech condition block. The presentation order of these intact and degraded speech blocks within each run was systematically varied and counterbalanced across participants to control for potential order effects. This counterbalancing approach is consistent with the methodology employed by Scott et al.^48^ There are four total runs. As in experiment 1, participants were told that they would listen to some fun audio clips and some clips that were distorted in a way that made it impossible to understand what the speaker was saying. They were instructed to listen attentively. Prior to the experiment, it was ensured that the volume level was sufficiently loud yet comfortable.

In the MD localizer task, participants completed a go/no-go task that was carefully adapted for their age group. To ensure task comprehension, a training session was conducted before the experiment, where the experimenter provided feedback and continued with practice trials until the child demonstrated a reliable understanding of the rules. The formal experiment consisted of four runs, each containing alternating “go” and ” go/no-go ” blocks separated by 10-s fixation periods. The design was structured to be engaging and cognitively manageable for toddlers. Each block began with a 3-s instruction screen followed by 12 trials lasting 12 s in total. In the simple “go” blocks, which served as the low-demand condition, participants were instructed to press a key for all presented animal pictures (e.g., both cats and dogs). In the more demanding “go/no-go” blocks, participants were required to execute a response for “go” stimuli (e.g., chicken images) but withhold their response for “no-go” stimuli (e.g., duck images). A study personnel monitored participant performance throughout the task to ensure continued engagement. This use of familiar animal stimuli, a simple response rule, and a clear contrast between a simple-response block and a response-inhibition block made the paradigm developmentally appropriate for probing cognitive control in our young cohort.

#### 2.2.3 Data acquisition

**Fig. 3:**
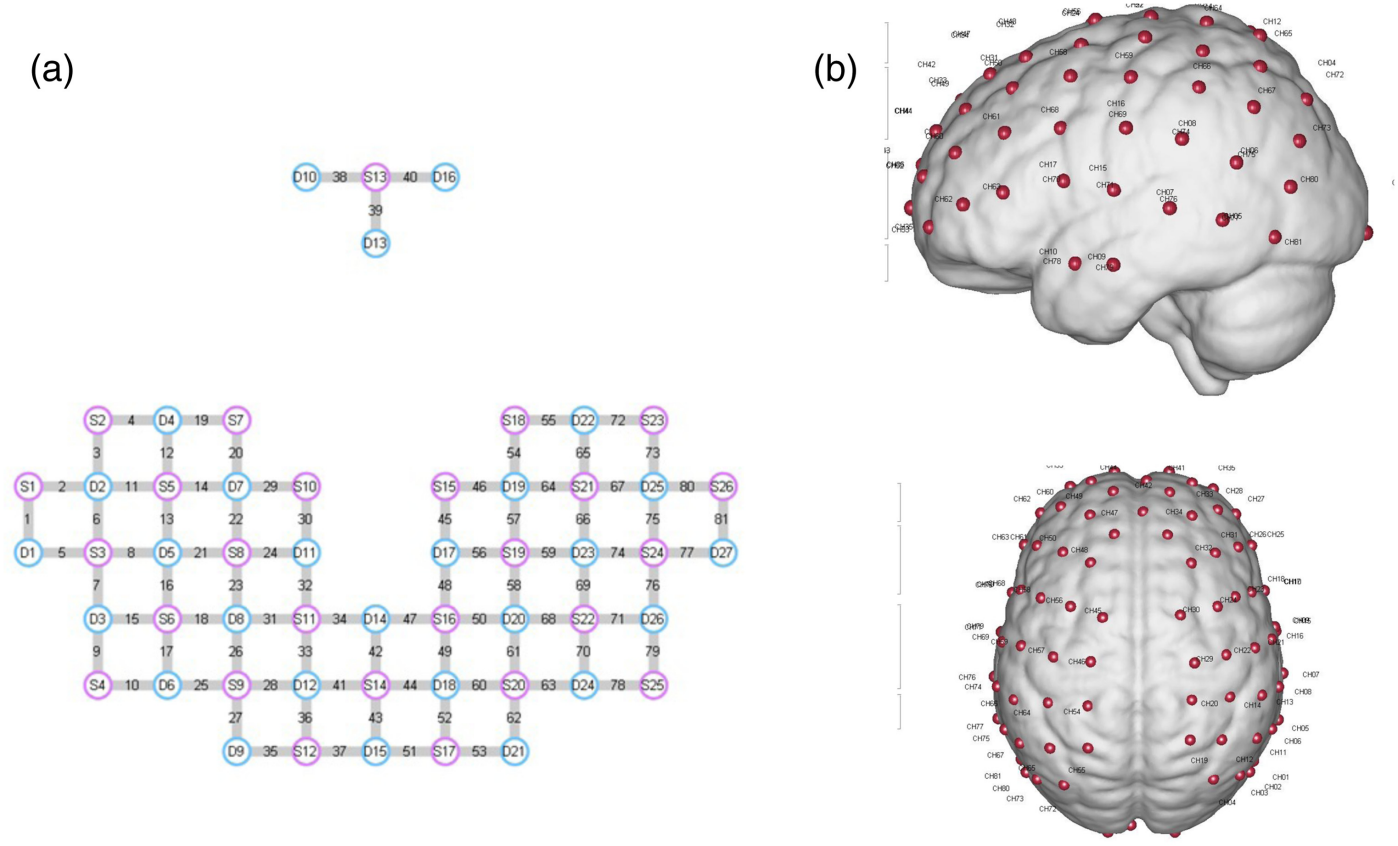
(a) Schematic representation of the fNIRS optode placement. Red circles indicate sources, blue circles indicate detectors, and yellow lines represent measurement channels. (b) Spatial registration result of the probe array. Red dots with numbers indicate channel positions on the MNI standard brain template.

fNIRS measurements were collected using a multichannel continuous-wave NirSmart fNIRS system (Danyang Huichuang Medical Equipment Co., Ltd., China). This system, designed for laboratory-based studies, utilized LED light sources emitting at wavelengths of 730, 808, and 850 nm. Light attenuation was recorded by avalanche photodiode detectors with a sampling rate of 11 Hz. A total of 26 sources and 27 probes constituted 81 measurement channels of 3 cm (see Fig. 3). The optodes were stabilized using a plastic holder and then affixed to participants’ heads over the frontal, temporal, occipital, and parietal lobes in the left and right hemispheres using custom headgear. When wearing the optode cap, it was ensured that the Cz point of the electrode cap coincided with the Cz point of the scalp surface measurement.

#### 2.2.4 Data preprocessing

Data were preprocessed in the same manner as experiment 1.

#### 2.2.5 Data analysis

Data exclusion criteria are the same as experiment 1. For each participant and channel, mean HbO2 concentration changes were calculated per trial. The analysis window started 5 s after stimulus onset and lasted until the end of the block window (17 s post-onset for the language localizer task or 12 s post-onset for the go/no-go task). This timing accounts for the HRF lag time commonly assumed in children’s NIRS research.^68^^,^^69^ Language and MD selective regions were identified, and mean HbO responses were extracted using individual fCOI approaches within the same search space (see Table 2 for probabilistic mapping of selected fNIRS channels to underlying cortical regions), following experiment 1’s methodology. Inclusion criteria required children to complete at least two runs per experiment, ensuring sufficient trials for the leave-one-run-out procedure. Additional exploratory analyses incorporating age as a fixed effect in the generalized linear mixed models are reported in the Supplementary Material. Same as experiment 1, Bonferroni correction was applied separately for each task (language versus cognitive control). In each task, the left and right IFG fCOIs formed a single family of two comparisons, yielding a family-wise threshold of α=0.025 (0.05/2).

**Table 2:** Probabilistic mapping of fNIRS channels to underlying cortical regions. The anatomical labeling and corresponding MNI coordinates for each source-detector pair were estimated using the NIRS-SPM software package. Percentages indicate the probability of the channel’s cortical projection falling within the specified Brodmann areas.

Channel	MNI coordinates	Anatomic label	Percentage (%)	Search space
17	77.67, 19.00, 17.33	6—pre-motor and supplementary motor cortex, 43—subcentral area, 44—pars opercularis, 45—pars triangularis Broca’s area, 48—retrosubicular area	51.44, 0.32, 42.49, 4.79, 0.96	RIFG
18	69.67, 20.33, 44.67	6—pre-motor and supplementary motor cortex, 9—dorsolateral prefrontal cortex, 44—pars opercularis	36.02, 9.20, 54.79	RIFG
25	69.33, 42.67, 13.33	45—pars triangularis Broca’s area, 46—dorsolateral prefrontal cortex	99.32, 0.68	RIFG
26	60.33, 42.67, 40.33	9—dorsolateral prefrontal cortex, 44—pars opercularis, 45—pars triangularis Broca’s area, 46—dorsolateral prefrontal cortex	0.81, 6.07, 75.30, 17.81	RIFG
27	59.67, 60.33, 5.67	10—frontopolar area, 45—pars triangularis Broca’s area, 46—dorsolateral prefrontal cortex	4.14, 7.52, 88.35	RIFG
56	46.00, 42.33, 59.67	10—frontopolar area, 45—pars triangularis Broca’s area, 46—dorsolateral prefrontal cortex	0.85, 4.68, 94.47	LIFG
57	33.00, 36.00, 73.00	44—pars opercularis, 45—pars triangularis Broca’s area, 46—dorsolateral prefrontal cortex	1.23, 90.95, 7.82	LIFG
59	33.67, 62.67, 50.67	45—pars triangularis Broca’s area, 46—dorsolateral prefrontal cortex	99.65, 0.35	LIFG
64	−57.67, −46.33, 79.33	6—pre-motor and supplementary motor cortex, 44—pars opercularis	33.45, 66.55	LIFG
66	−70.33, 18.33, 39.00	6—pre-motor and supplementary motor cortex, 44—pars opercularis, 45—pars triangularis Broca’s area, 48—retrosubicular area	49.49, 31.99, 0.67, 17.85	LIFG

## 3 Results

### 3.1 Experiment 1

No participants were excluded based on our criteria for channel rejection and fCOI rejection. For the subsequent analyses, we included all data after removing the excluded noisy channel and any runs where noisy channels comprised more than one-third of the search space. Table 3 provides a summary of the available runs across participants for different fCOI types (see the distribution of identified channels for each type of fCOI in Figs. S4–S8 in the Supplementary Material).

**Table 3:** Summary of the available runs across participants for different fCOI types.

fCOI type	Total LAN subjects included	Mean LAN runs (SDs)	Total MD subjects included	Mean MD runs (SDs)
LAN LIFG	20	6.95 (0.683)	20	3.85 (0.489)
LAN RIFG	20	7.15 (0.3663)	20	3.85 (0.489)
MD LIFG	20	7.10 (0.4472)	20	3.90 (0.4472)
MD RIFG	20	7.15 (0.3663)	20	3.90 (0.4472)

**Fig. 4:**
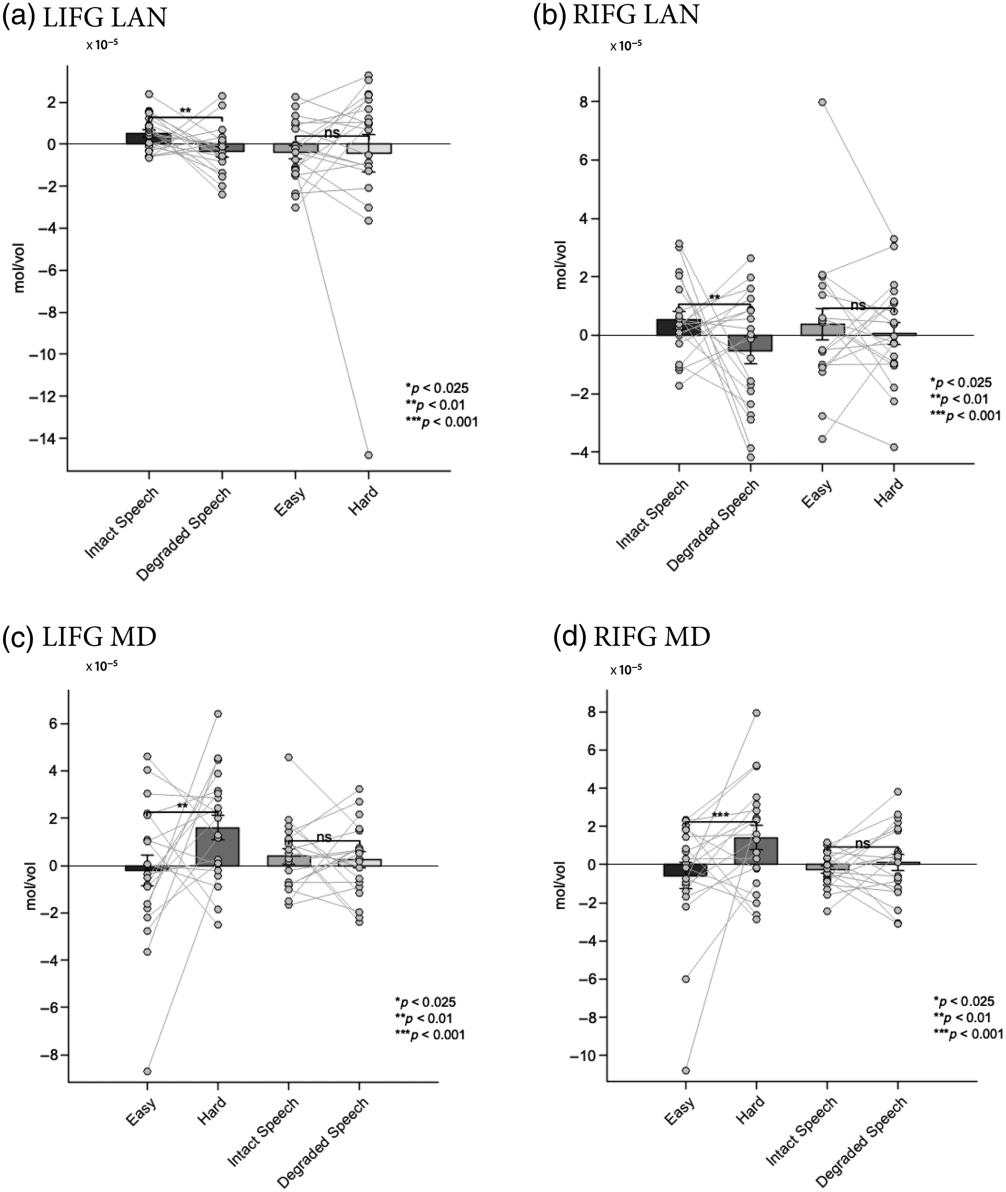
Results from experiment 1: mean HbO2 responses in the selected fCOIs. (a) Left hemisphere language fCOI responses showing significant selectivity for intact versus degraded speech (p=0.005) and no significant modulation by cognitive demand (p=0.940). (b) Right hemisphere language fCOI responses showing significant selectivity for intact versus degraded speech (p=0.008) and no significant modulation by cognitive demand (p=0.665). (c) Left hemisphere MD fCOI responses showing significant sensitivity to cognitive demand (p<0.001) and no significant difference between speech conditions (p=0.061). (d) Right hemisphere MD fCOI responses showing significant sensitivity to cognitive demand (p<0.001) and no significant difference between speech conditions (p=0.325). Error bars represent the standard error of the mean.

The language fCOIs showed clear selectivity for language processing. In both hemispheres, these channels demonstrated significantly stronger responses to intact versus degraded speech, with all findings surviving Bonferroni correction for two comparisons (α=0.025). The LIFG language fCOI showed a significant effect of condition (β=−9.06e−06, SE=3.23e−06, t=−2.81, p=0.005), with higher activation for intact speech. Similarly, the RIFG language fCOI exhibited a significant preference for intact speech (β=−1.05e−05, SE=3.94e−06, t=−2.67, p=0.008). Importantly, when tested on the spatial working memory task, neither the left nor right language fCOIs showed significant modulation by cognitive demand after Bonferroni correction (LIFG: β=4.44e−07, SE=5.85e−06, t=0.08, p=0.940; RIFG: β=−2.46e−06, SE=5.68e−06, t=−0.43, p=0.665). This pattern suggests that the language fCOIs are specifically selective for language processing and do not respond systematically to increased cognitive load in nonlinguistic tasks [Figs. 4(a) and 4(b)].

**Fig. 5:**
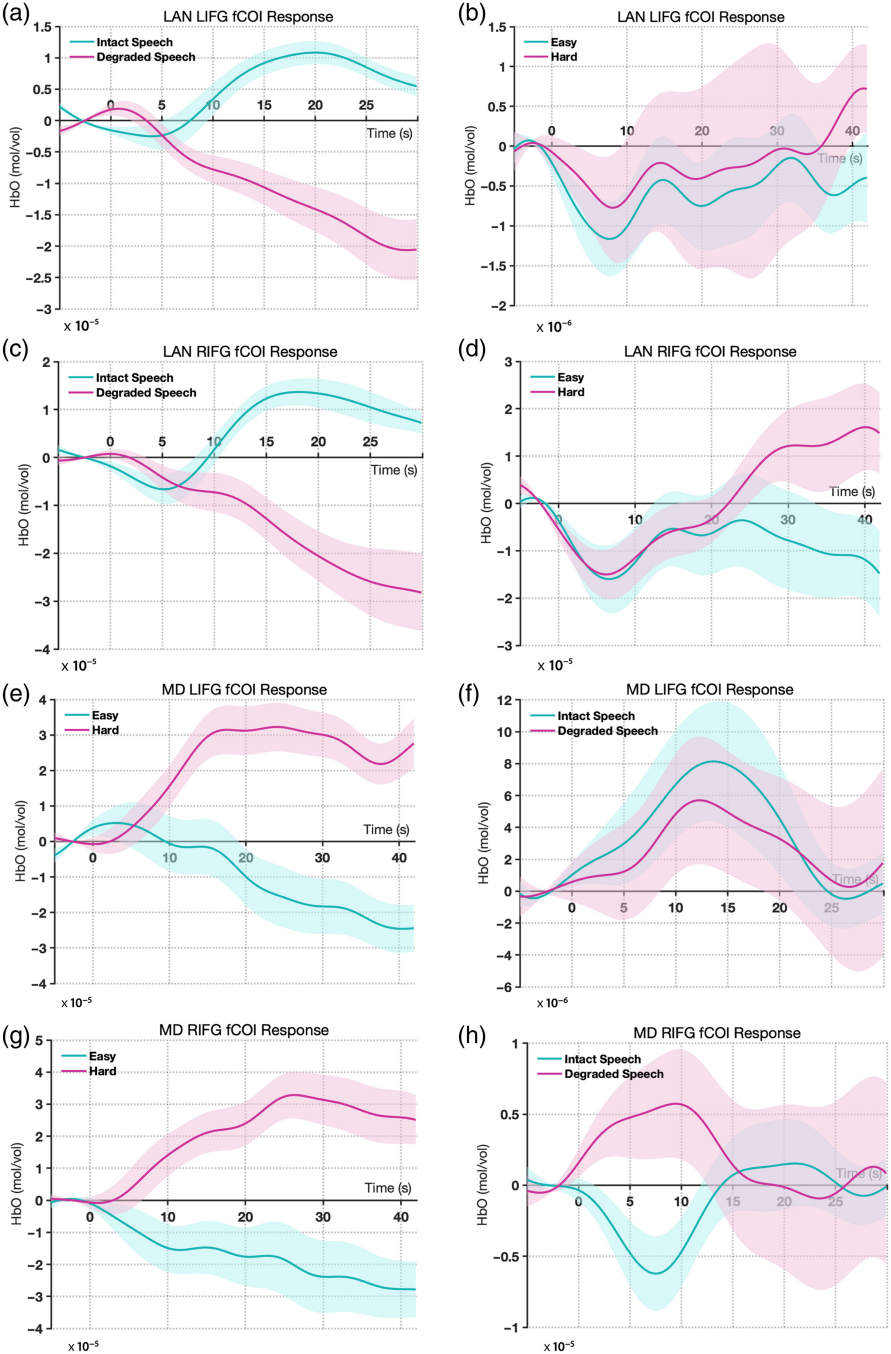
Hemodynamic time courses in adult fCOIs (experiment 1). Grand-average change in oxygenated hemoglobin (HbO) concentration is shown for each functionally defined region of interest (fCOI) type. Solid lines represent the mean response, and the shaded areas represent the standard error of the mean (SEM). Time is in seconds relative to block onset. (a), (c) Language-selective fCOIs in both the left (LIFG) and right (RIFG) inferior frontal gyrus showed a selective response to intact versus degraded speech. (b), (d) These same language fCOIs did not show a differential response to the easy versus hard conditions of the spatial WM task. (e), (g) Conversely, multiple-demand (MD) fCOIs in the LIFG and RIFG showed a selective response to the hard versus easy conditions. (f), (h) These MD fCOIs did not respond differentially to intact versus degraded speech, demonstrating a clear double dissociation between the language and multiple-demand networks in adults.

The MD fCOIs demonstrated a complementary pattern of activation. Both left and right MD fCOIs showed significant sensitivity to cognitive demand in the spatial working memory task, with all findings surviving Bonferroni correction for two comparisons (α=0.025). The LIFG MD fCOI exhibited stronger activation for harder versus easier conditions (β=1.88e−05, SE=4.82e−06, t=3.90, p<0.001), as did the RIFG MD fCOI (β=2.04e−05, SE=5.64e−06, t=3.62, p<0.001). By contrast, when tested on the language task, neither MD fCOI showed significant differentiation between intact and degraded speech after Bonferroni correction (LIFG: β=−7.28e−06, SE=3.87e−06, t=−1.88, p=0.061; RIFG: β=3.61e−06, SE=3.66e−06, t=0.99, p=0.325). This finding indicates that the MD network responds selectively to cognitive demand but not to linguistic properties per se [Figs. 4(c) and 4(d)]. The corresponding hemodynamic time courses for all conditions are shown in Fig. 5 for HbO and Fig. S11 in the Supplementary Material for HbR. The analysis of deoxyhemoglobin is detailed in the Supplementary Material.

These results demonstrate a double dissociation between the language and MD networks, with language fCOIs showing selectivity for linguistic processing but not cognitive demand, and MD fCOIs showing the opposite pattern.

### 3.2 Experiment 2

Table 4 provides a summary of the included participants and available runs across participants for different fCOI types based on the criteria for fCOI rejection we described in experiment 1 (see the distribution of identified channels for each type of fCOI in Figs. S1–S4 in the Supplementary Material).

**Table 4:** Summary of the available runs across participants for different fCOI types.

fCOI type	Total LAN subjects included	Mean LAN runs (SDs)	Total MD subjects included	Mean MD runs (SDs)
LAN LIFG	22	3.54 (0.510)	21	2.24 (0.436)
LAN RIFG	22	3.50 (0.597)	17	2.23 (0.437)
MD RIFG	22	3.50 (0.597)	22	2.27 (0.457)
MD LIFG	21	3.38 (0.74)	21	2.24 (0.539)

**Fig. 6:**
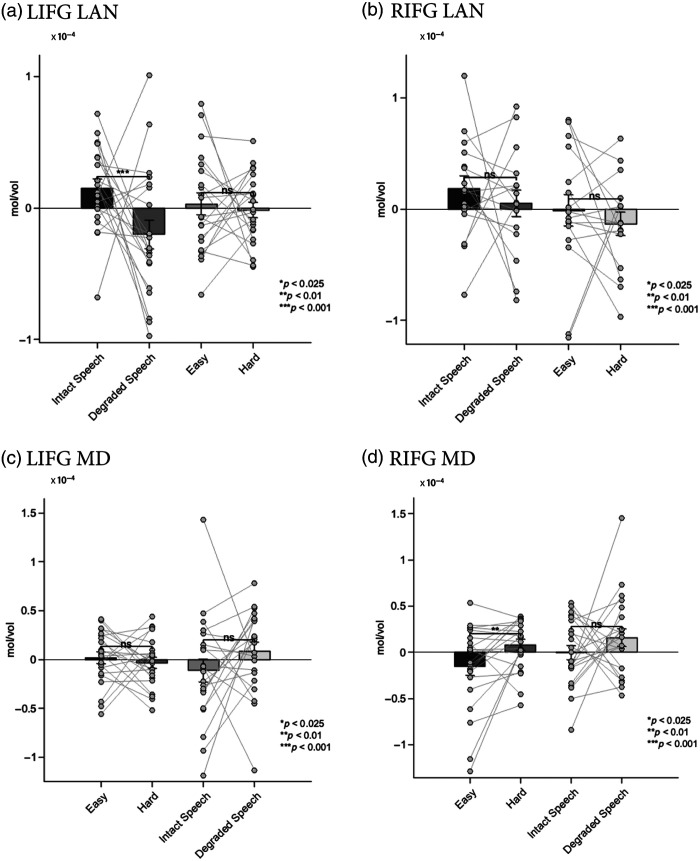
Results from experiment 2: Mean HbO2 responses in the selected fCOIs (a) Left hemisphere language fCOI responses showing significant selectivity for intact versus degraded speech (p=0.00057) and no significant modulation by cognitive demand (p=0.652). (b) Right hemisphere language fCOI responses showing no significant selectivity for speech conditions (p=0.925) and no significant modulation by cognitive demand (p=0.937. (c) Left hemisphere MD fCOI responses showing no significant sensitivity to cognitive demand (p=0.305) but a significant preference for degraded speech (p=0.038). (d) Right hemisphere MD fCOI responses showing significant sensitivity to cognitive demand (p=0.0061) and marginal preference for degraded speech (p=0.221). Error bars represent the standard error of the mean.

Analysis of the language fCOIs in children showed a partially overlapping pattern with adults. The LIFG language fCOI maintained significant selectivity for language processing, showing stronger responses to intact versus degraded speech (β=−3.83e−05, SE=1.09e−05, t=−3.52, p=0.00057) after Bonferroni correction for two comparisons (α=0.025). However, unlike adults, the RIFG language fCOI did not show significant differentiation between intact and degraded speech after Bonferroni correction (β=1.09e−06, SE=1.16e−05, t=0.09, p=0.925). Consistent with the adult findings, neither language fCOI showed significant modulation by cognitive demand in the go/no-go task after Bonferroni correction (LIFG: β=−4.24e−06, SE=9.37e−06, t=−0.45, p=0.652; RIFG: β=8.14e−07, SE=1.03e−05, t=0.08, p=0.937). This suggests that even in children, the language fCOI we identified maintains specificity for language processing over general cognitive demands [Figs. 6(a) and 6(b)].

**Fig. 7:**
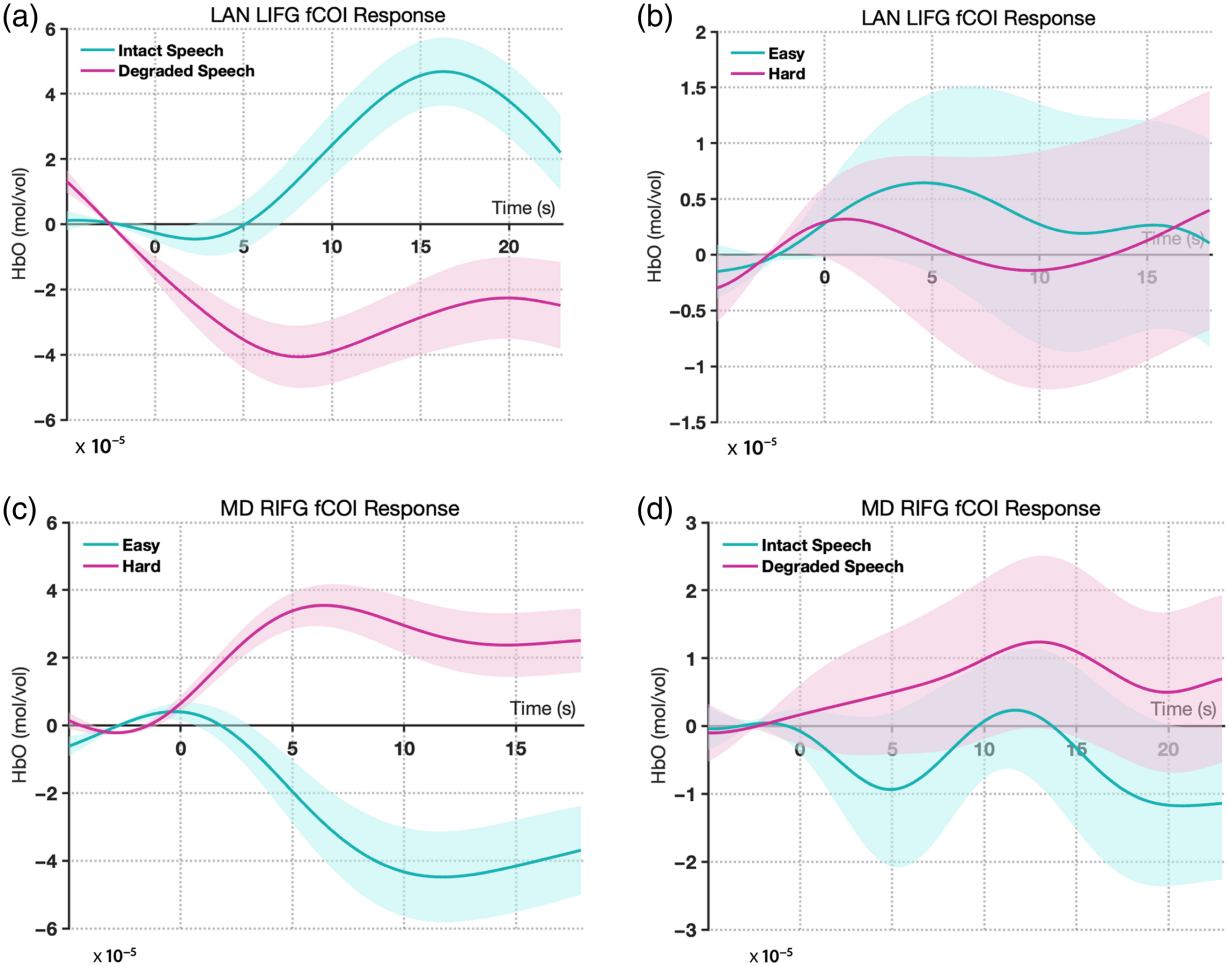
Hemodynamic time courses in toddler fCOIs (experiment 2). Grand-average change in oxygenated hemoglobin (HbO) concentration is shown. Solid lines represent the mean response, and the shaded areas represent the standard error of the mean (SEM). Time is in seconds relative to block onset. (a) The language-selective fCOI in the left inferior frontal gyrus (LIFG LAN) showed a stronger response to intact speech compared to degraded speech, and (b) this same region did not respond differentially to the cognitive demand of the go/no-go task. (c) The multiple-demand fCOI in the right inferior frontal gyrus (RIFG MD) showed a stronger response to the hard (go/no-go) condition compared with the easy (go-only) condition, and (d) this same region was insensitive to the linguistic contrast, demonstrating an early-emerging functional dissociation.

The MD fCOI we identified in children showed a more complex pattern than expected. Behavioral performance confirmed the task difficulty manipulation, with participants showing higher accuracy on go trials (M=81%, SD=12%) compared with no-go trials (M=73%, SD=15%; t(21)=2.25, p=0.035). Only the right MD fCOI displayed higher sensitivity to cognitive demand in the go/no-go task after Bonferroni correction (significantly stronger responses to the harder versus easier condition; RIFG: β=2.43e−05, SE=8.68e−06, t=2.80, p=0.0061). By contrast, the left MD fCOI did not show a significant difference between these conditions after Bonferroni correction (LIFG: β=−7.74e−06, SE=7.51e−06, t=−1.03, p=0.305). When tested for specificity on the language task, neither MD fCOI showed a significant response after Bonferroni correction. The left MD fCOI showed a nonsignificant trend toward greater activation for degraded speech (β=2.22e−05, SE=1.06e−05, t=2.10, p=0.038), whereas the right MD fCOI was clearly insensitive to the linguistic manipulation (β=1.35e−05, SE=1.10e−05, t=1.23, p=0.221). These findings suggest that only the right MD fCOI displays the characteristic MD network profile of responding to cognitive control demands but not to linguistic properties [Figs. 6(c) and 6(d)]. The corresponding hemodynamic time courses for all conditions are shown in Fig. 7 for HbO and Fig. S12 in the Supplementary Material for HbR. The results for deoxyhemoglobin are detailed in the Supplementary Material.

## 4 Discussion

Our study investigates how the brain supports language processing in early childhood, focusing on whether neural circuits are specialized for language or shared with other cognitive domains. By employing fNIRS with an individual functional channel of interest approach, we assessed both adults and toddlers on language and cognitive control tasks to seek evidence for distinct functional profiles in the left and right inferior frontal regions. Our adult findings validated the fCOI method by replicating well-established dissociations between language-specific and domain-general processes, providing a foundation for investigating the developmental period. Our toddler results suggest that left-hemisphere language selectivity emerges early and remains robust, whereas right-hemisphere organization shows signs of ongoing maturation.

In line with adult findings and recent work in older children,^29^^,^^70^ toddlers demonstrated robust language selectivity in the left IFG. The language fCOI within this area showed specific responses to intact versus degraded speech, mirroring the adult pattern, while remaining insensitive to the manipulation of cognitive demands in the go/no-go task. This supports the hypothesis of early domain specificity in language processing, as the language-selective region within the left IFG region exhibited both selectivity and specificity for language.^36^^,^^71^ Our toddler data align with evidence that even very young children recruit the left frontal cortex for high-level linguistic processing.^33^^,^^35^^,^^72^^,^^73^ Moreover, our findings revealed a developmental asymmetry in language processing between hemispheres, specifically within the IFG. Although our adult participants showed bilateral IFG fCOIs with selectivity for meaningful speech, toddlers did not exhibit statistically reliable language responses in the RIFG language fCOI. This asymmetry aligns with previous developmental research and recent findings in older children. Hiersche et al.,^29^ using a similar speech localizer task comparing sentences to acoustically degraded speech, also found no significant language selectivity in the right IFG. Early in development, infant studies have documented limited right-hemisphere sensitivity to language in speech versus control conditions.^33^^,^^72^ A developmental shift occurs later: children ages 5 to 11 show right hemisphere engagement, particularly during complex linguistic tasks requiring greater syntactic processing or elevated linguistic demands.^74^^–^^76^ Our observation of strong LIFG but nonsignificant RIFG selectivity in toddlers (ages 2 to 4) suggests that RIFG language specialization follows a prolonged developmental trajectory. Although LIFG supports core linguistic computations from early development, RIFG may serve complementary functions that become increasingly important as language abilities mature. Studies suggested RIFG involvement in prosodic processing, pragmatic aspects of language, and discourse-level comprehension.^77^^,^^78^ The absence of RIFG language selectivity in toddlers suggests that these higher-level linguistic functions develop later, potentially aligning with children’s growing ability to process complex narrative^79^ and social aspects of language.^80^ These findings support a developmental model where left-hemisphere language selectivity emerges early and remains stable, whereas right-hemisphere language involvement develops gradually throughout childhood.^74^^,^^81^

With regard to domain-general processing, our findings revealed a distinct pattern in toddlers from adults: only the right MD fCOI showed selective sensitivity to increased cognitive demands during the go/no-go task. These MD fCOIs demonstrated functional profiles that contrasted with language fCOIs—whereas language regions responded selectively to intact versus degraded speech but showed no sensitivity to cognitive demands, MD fCOIs exhibited the opposite pattern. Importantly, only the right MD fCOI displayed the characteristic MD network profile of responding to cognitive control demands but not to linguistic properties. This toddler-specific pattern diverges from established findings in both adults and older children (∼7 to 12 years). Adults show bilateral MD network responses to escalating task difficulty, and older children demonstrate bilateral prefrontal recruitment during inhibitory control tasks.^82^^–^^85^ The developmental differences likely reflect the ongoing structural and functional specialization of the MD network in early childhood. Our findings of right MD responsiveness in toddlers, coupled with previous fNIRS and fMRI studies, suggest that very young children’s prefrontal cortex involvement in executive tasks may initially manifest in right hemisphere regions compared to older groups.^86^^,^^87^ This aligns with evidence of protracted maturation for left frontal functions.^82^^,^^84^^,^^88^ The left MD fCOI’s lack of modulation by task difficulty suggests that an adult-like MD network component may not yet be established in the left IFG in our sample. This developmental pattern is further corroborated by recent work from Schettini et al.^30^ who similarly found that the left IFG in children did not show significant selectivity for cognitive demand. Together, these findings support previous research indicating ongoing development of prefrontal involvement in domain-general control,^30^^,^^81^^,^^83^ with bilateral integration emerging later in development as children increasingly recruit both hemispheres for executive tasks.

Our use of the fCOI approach addressed several methodological challenges in fNIRS studies. First, it circumvents the limitations of the “reverse inference” problem, which plagues many fNIRS studies that rely on inferring cognitive functions solely from anatomical correspondence with prior findings. This practice is problematic as the same anatomical location may support different functions across individuals,^89^ particularly in developmental research where variations in head size, brain organization, and probe placement complicate anatomical localization.^42^ Our fCOI approach addresses these limitations by identifying regions based on functional properties rather than assumed anatomical locations, effectively accommodating individual variability in neural functional anatomy across development. Second, it provided enhanced statistical power while controlling for multiple comparisons, which is crucial when working with limited developmental data.^41^ Third, the implementation of the same localizer task used by fMRI studies^7^^,^^29^^,^^70^ allowed for direct comparison of properties of specific functional regions over developmental stages and imaging modalities. Our successful identification of functionally distinct networks in both adults and toddlers demonstrates the robustness of this approach across developmental stages.

Our experimental design also addressed several methodological challenges. The implementation of a leave-one-run-out cross-validation procedure for fCOI selection avoided the “double dipping” issue in neuroimaging studies,^90^ enhancing the reliability of our findings. By employing passive language tasks contrasting meaningful versus degraded speech, we controlled for confounding effects of task difficulty while maintaining prosodic and rhythmic features.^48^ This paradigm, extensively validated in adult studies across different languages, allowed us to specifically probe the processing of high-level linguistic features such as semantics and syntax.^49^ The careful selection of age-appropriate tasks for probing the MD network—spatial working memory for adults and Go/No-go for children—enabled us to examine domain-general processing while accommodating developmental constraints. By comparing responses to both linguistic and nonlinguistic tasks within the same participants, we directly assessed the specificity of these networks to language processing.

Despite these methodological strengths, several limitations warrant discussion. First, like most fNIRS experiments, our study faced challenges of limited and noncontinuous cortical coverage. Although our probe arrangement provided good coverage of bilateral inferior frontal regions, some cortical areas may have fallen into measurement “blind spots.”^91^ Second, we did not have access to short-separation channels in our study. Short-separation channels are efficient in accounting for and removing superficial hemodynamic signals originating from the scalp and skull, thereby isolating the cortical activity of interest.^92^^,^^93^ The absence of short-separation channels may have led to contamination of our signals by systemic physiological artifacts. Although we have applied the global regression method to filter out systemic physiological artifacts,^57^ systemic physiological artifacts may still potentially affect the specificity and accuracy of our findings. Third, we also noted a negative deflection of the HbO signal in some conditions. This may be the consequence of the analysis pipeline and does not necessarily indicate physiological deactivation. Data-driven noise correction, such as the principal component spatial filter used here, removes widespread systemic artifacts by design, which can shift the mean response in lower-activity conditions below zero.^57^^–^^59^ Furthermore, because fNIRS measures are inherently relative, any minor elevation in the pre-stimulus baseline from an incomplete hemodynamic return can also produce an apparent negative change. Critically, our conclusions are based on the contrast between conditions, a relative comparison unaffected by these absolute signal shifts. Thus, this feature does not alter our central findings regarding the functional dissociation of the language and multiple-demand networks. Fourth, different cognitive tasks were used for adults and children. Although the adult MD regions identified by the spatial working memory localizer have been demonstrated to respond to a wide variety of cognitive demands, our use of a single executive function task in toddlers can only provide direct evidence for engagement in inhibitory control. The “domain-general” nature of the network we localized in toddlers is an inference based on the adult literature.^22^ Consequently, we must be cautious in concluding that the regions we identified in toddlers via a go/no-go task are truly “domain-general” in the same broad sense as the adult MD network. Their responsiveness to other cognitive challenges (e.g., working memory or arithmetic) remains to be tested. Nonetheless, the central finding of this study—the clear functional dissociation between a region sensitive to linguistic processing and a network sensitive to nonlinguistic cognitive effort—remains robust and provides strong support for the early specialization of core language circuits. Fifth, although our peak-channel selection approach within predefined anatomical regions of interest was optimized for our specific hypotheses, alternative approaches might be valuable for different research questions. In addition, we did not examine behavioral metrics of language development, which limits our ability to differentiate between changes in language selectivity due to maturation versus language skill development.^94^ Finally, our focus was primarily on the inferior frontal cortex, which constrained our ability to fully characterize both the MD network and the language-selective network. Although the IFG is a critical hub for domain-general cognitive control^9^ and language processing,^10^ the broader language network encompasses distributed temporal, parietal, and superior frontal regions. By restricting our measurements to the IFG, we may have overlooked crucial activity in other regions of the language network (e.g., the posterior temporal lobe) that could further elucidate the transition from domain-general to domain-specific processes in language development. Consequently, caution is warranted when generalizing our findings to the broader MD and language networks.

Future research should extend these findings in the following directions. First, longitudinal studies beginning at earlier ages^5^^,^^72^ are needed to clarify whether language networks transition from domain-general to domain-specific functioning over time. Second, studies should examine neural specialization at key language acquisition milestones by collecting brain measures when children reach specific linguistic achievements (e.g., first words, word combinations, complex sentences). This milestone-based approach^7^^,^^94^ would illuminate how maturing brain circuits support children’s emerging linguistic abilities. Third, to bridge the methodological gap inherent in comparing distinct age groups, future studies should employ tasks that can be parametrically adjusted for difficulty across a wide age range.

To conclude, using the methodologically improved fCOI approach with fNIRS, we replicated well-established patterns of bilateral selectivity and specificity in adult participants during linguistic and cognitive control tasks. In toddlers, we found early specialization of the language-selective component within LIFG, whereas the homologous right hemisphere region had not yet developed language selectivity. On cognitive control tasks, toddlers showed adult-like patterns in the within RIFG only. Notably, the selected MD fCOI in toddler’s LIFG only showed engagement with degraded speech rather than the broader domain-general profile seen in adults, suggesting ongoing maturation of domain-general circuits. These findings advance our understanding of how specialized brain circuits for language emerge during early childhood and establish the utility of the fCOI approach for studying functional organization in the developing brain.

## Supplementary Material

10.1117/1.NPh.12.3.035012.s01

## Data Availability

The data that support the findings of this study are available from the corresponding author upon reasonable request. The data are not publicly available due to privacy or ethical restrictions involving data from minor participants.
