# Supplementary material for: Functional specialization for language processing in inferior frontal regions during early childhood: evidence from functional near-infrared spectroscopy individual functional channels of interest approach
Source: Neurophotonics. 2025 Sep 13;12(3):035012. doi: 10.1117/1.NPh.12.3.035012 (PMC12432833; doi:10.1117/1.NPh.12.3.035012)
Supplement: Supplementary file 1 [file NPh_012_035012_SD001.pdf]

Supplementary material

Table of Contents

1. Stimulus Material

1.1 Adult

- **1.1.1 Intact Speech Condition** - 24 stories
- **1.1.2 Degraded Speech Condition** - 8 stories

1.2 Children

- **1.2.1 Intact Speech** - 4 stories
- **1.2.2 Degraded Speech** - 4 stories

2. The Distribution of Identified Channels for Each Type of fCOI

2.1 Child Brain Region Distribution

- Figure S1: Child LAN Left
- Figure S2: Child LAN Right
- Figure S3: Child MD Left
- Figure S4: Child MD Right

2.2 Adult Brain Region Distribution

- Figure S5: Adult LAN Left
- Figure S6: Adult LAN Right
- Figure S7: Adult MD Left
- Figure S8: Adult MD Right

3. Supplementary Results: Deoxygenated Hemoglobin (HbR) Analyses

3.1 Adults

- **3.1.1 Language fCOIs**
  - LIFG language fCOI results
  - RIFG language fCOI results
- **3.1.2 MD fCOIs**
  - LIFG MD fCOI results
  - RIFG MD fCOI results

3.2 Children

- **3.2.1 Language fCOIs**

  - LIFG language fCOI results
  - RIFG language fCOI results
- **3.2.2 MD fCOIs**
  - LIFG MD fCOI results
  - RIFG MD fCOI results

3.3 HbR Results Figures

- Figure S9: Adult HbR responses (no significant effects after correction)
- Figure S10: Toddler HbR responses showing various significant effects
- Figure S11: Hemodynamic time courses in adult fCOIs
- Figure S12: Hemodynamic time courses in toddler fCOIs

4. Exploratory Analysis: Age-related Effects in Experiment 2

4.1 MD fCOI Analysis

- Main effect of cognitive condition
- Age effects and interactions

4.2 Language fCOI Analysis

- Marginal age effects
- Condition-by-age interactions

4.3 Developmental Implications

- Discussion of foundational functional organization
- Methodological considerations for future studies

Stimulus material

Adult

Intact speech condition (adapted from Gweon et al (2012) into Mandarin Chinese)

1. 一天，海盗告诉了小明一个关于宝藏的秘密，说他觉得宝藏就埋在小明家。小明相信了他，在自己家的后面挖了一个大洞，但是他没有找到宝藏。小明意识到海盗其实不知道宝藏在哪。English translation :One day, a pirate told Xiao Ming a secret about a treasure, saying he thought the treasure was buried at

Xiao Ming's home. Xiao Ming believed him and dug a big hole behind his house, but he didn't find any treasure. Xiao Ming realized that the pirate actually didn't know where the treasure was.

2. 今天，小亮为他的朋友小明准备了一个生日派对。小亮邀请小明来打篮球。小亮不想让小明知道派对的事，所以他没有和小明说生日快乐。小明以为小亮忘记了今天是他的生日，他非常难过。English translation :Today, Xiao Liang prepared a birthday party for his friend Xiao Ming. Xiao Liang invited Xiao Ming to play basketball. Xiao Liang didn't want Xiao Ming to know about the party, so he didn't say happy birthday to him. Xiao Ming thought Xiao Liang had forgotten his birthday, and he was very sad.
3. 渔夫和他的妻子住在一个破旧的棚屋里。有一天，他捕到了一只会说话的鱼，他非常惊讶。鱼答应可以帮他实现一个愿望。渔夫的妻子想要一个新房子，但她不相信一条鱼可以满足他们这个愿望。English translation :A fisherman and his wife lived in a rundown hut. One day, he caught a talking fish, which surprised him greatly. The fish promised to grant him one wish. The fisherman's wife wanted a new house, but she didn't believe a fish could fulfill their wish.
4. 有一天，两个女孩在森林里散步。她们看到一个胡子很长的怪物躺在石头旁边。女孩们以为怪物的胡子被石头压住了，她们就剪掉了怪物的胡子想救他。 但是怪物觉得她们毁了它的胡子，他很生气。English translation :One day, two girls were walking in the forest. They saw a monster with a long beard lying beside a rock. The girls thought the monster's beard was trapped under the rock, so they cut off the monster's beard to save him. But the monster felt they had ruined his beard, and he was very angry.
5. 离开营地前，小红画了一张自己的画像送给她的好朋友小张。小张看到这副画的时候不知道这是小红的画像，她开玩笑地在画上写了几个字——“可怕的怪物”，然后被小红看到了，她就很生气。English translation :Before leaving the camp, Xiao Hong drew a self-portrait and gave it to her good friend

Xiao Zhang. When Xiao Zhang saw the drawing, she didn't know it was Xiao Hong's portrait, and she jokingly wrote a few words on it—"terrible monster"—which Xiao Hong saw, and she became very angry.

6. 有一天，世界上所有人都变成了紫色。人们互相打量，想知道自己是不是在做梦。每个人都感到震惊，即使是最聪明的科学家也不知道发生了什么。但是小明一点也不惊讶，他知道这个秘密。English translation :One day, everyone in the world turned purple. People looked at each other, wondering if they were dreaming. Everyone was shocked, even the smartest scientists didn't know what had happened. But Xiao Ming wasn't surprised at all; he knew the secret.
7. 小明的妈妈让他去湖边捉一条鱼当作今天的晚餐。小明害怕一个人去湖边，他觉得湖里有水怪。妈妈知道小明说的水怪其实只是他自己的倒影，她告诉小明可以试着对那个水怪笑一笑。English translation :Xiao Ming's mother asked him to go to the lake to catch a fish for dinner. Xiao Ming was afraid to go to the lake alone, thinking there was a water monster in the lake. His mother knew that the water monster was actually just his reflection, so she told Xiao Ming to try smiling at the monster.
8. 男孩们很担心，爸爸希望他们早点回家，但他们已经很晚了还没回去。小明在地图上找到了一条小路，但树挡住了小路的入口。男孩们知道他们很容易走过，所以他们很仔细地看路。English translation :The boys were worried. Their father wanted them to come home early, but they were already late and hadn't returned. Xiao Ming found a shortcut on the map, but trees blocked the entrance to the path. The boys knew they could easily get through, so they looked at the path very carefully.
9. 有一天晚上，小红的父母带她去奶奶家过夜。因为奶奶年纪很大了，所以早早就上床睡觉了。于是小红开始在奶奶的衣柜里玩。在衣柜的最里面，有一双漂亮的红鞋子，小红穿上了这双鞋。English translation :One evening, Xiao Hong's parents took her to stay overnight at her grandmother's house. Since

grandmother was very old, she went to bed early. So Xiao Hong started playing in grandmother's closet. At the very back of the closet, there was a pair of beautiful red shoes, and Xiao Hong put them on.

10. 小红和小张是学校足球队的队员。小红通常打进攻，在场上跑来跑去然后射门。小张是球队的守门员，当对方球员射门时，她会把球接住。今天学校有一场重要的比赛，但小张却生病了。English translation :Xiao Hong and Xiao Zhang are members of the school soccer team. Xiao Hong usually plays offense, running around on the field and shooting goals. Xiao Zhang is the team's goalkeeper, catching the ball when opposing players shoot. Today there is an important game at school, but Xiao Zhang is sick.
11. 从前，有一个女孩和她的弟弟去田野里采花。那天天气很热，弟弟非常口渴。在回家的路上，他喝了一个水坑里的水，但是那个水坑被下过魔咒。喝完水后，男孩变成了一只小山羊。English translation :Once upon a time, a girl and her little brother went to the fields to pick flowers. The weather was hot that day, and the brother was very thirsty. On their way home, he drank water from a puddle that had been cursed. After drinking the water, the boy turned into a little goat.
12. 从前有一个音乐家，她的笛子吹得非常好。每当她吹笛子的时候，所有人都会情不自禁地开始跳舞。直到她停止演奏，人们才会停下来。一天晚上，有个小偷去了音乐家的家里偷东西。English translation :There once was a musician who played the flute very well. Whenever she played her flute, everyone would involuntarily start dancing. Only when she stopped playing would people stop. One night, a thief went to the musician's house to steal things.
13. 小紫和小红是双胞胎，她们俩和父母一起住在大城市里。放暑假的时候，她们去爷爷家待了两个星期。她们住在爷爷湖边的房子里。每天早上，爷爷都会带着女孩们去游泳或划船。English translation :Xiao Zi and Xiao Hong are twins who live with their parents in a big city. During summer vacation, they spent two weeks at their grandfather's house. They stayed in grandfather's house

by the lake. Every morning, grandfather would take the girls swimming or boating.

14. 陈妈和她的儿子小杰住在一条小河附近。小杰是一个渔民，他平常会去河里捕鱼。小杰的父亲教过他捕鱼的方法，但他已经去世了。小杰卖鱼赚的钱，勉强能让他和陈妈吃饱饭。English translation :Mrs. Chen and her son Xiao Jie lived near a small river. Xiao Jie was a fisherman who would usually go fishing in the river. Xiao Jie's father had taught him how to fish, but he had passed away. The money Xiao Jie earned from selling fish barely allowed him and Mrs. Chen to have enough to eat.
15. 老刘是个满脸皱纹的老农民，他总是穿着皱巴巴的灰色旧衣服和靴子。小王是老刘住在城里的侄子。一天，小王穿上了老刘的那双大靴子。因为靴子太大了，小刘在靴子里消失了。English translation :Old Liu was a wrinkled old farmer who always wore wrinkled gray old clothes and boots. Xiao Wang was Old Liu's nephew who lived in the city. One day, Xiao Wang put on Old Liu's big boots. Because the boots were too big, Xiao Liu disappeared inside the boots.
16. 从前有一个贫穷的猎人，他在鼻子下面挂了一根又大又油腻的香肠。这根香肠很长，从猎人的鼻子一直下垂到他的脚趾头上。但不管他怎么用力拽那根香肠，拉它甚至割它，香肠都一动不动。English translation :Once there was a poor hunter who had a large, greasy sausage hanging under his nose. This sausage was so long that it hung from the hunter's nose all the way down to his toes. But no matter how hard he pulled the sausage, tugged at it, or even tried to cut it, the sausage wouldn't budge.
17. 一天，一只麻雀停在了一棵长得很奇怪的大树上，还吃了一个果子。这是一棵拥有神奇力量的魔法树。春天的时候，这只麻雀下了三个蛋。其中两个小麻雀很快就破壳而出了，但第三个蛋一直没有动静。English translation :One day, a sparrow landed on a strange-looking big tree and ate a fruit. This was a magic tree with magical powers. In spring, the sparrow laid three eggs. Two baby sparrows hatched quickly, but the third egg showed no movement.

18. 在一个村子里，有两个房子建在了一起。其中一个房子是用木头建的，又高又窄；另一个房子是用砖头建的，又矮又宽。一天晚上，村里面了一场暴雨。到了早上，只有砖头房子没倒下。English translation :In a village, there were two houses built next to each other. One house was made of wood, tall and narrow; the other house was made of bricks, short and wide. One night, the village experienced a heavy rainstorm. By morning, only the brick house was left standing.
19. 在一个魔法小镇里，每天会下三次雨雪。一次是在早上，一次在中午，一次在晚上。但小镇里下的不是普通的雨雪，而是果汁雨或者是土豆泥雪之类的东西。English translation :In a magical town, it rains or snows three times every day. Once in the morning, once at noon, and once in the evening. But the town doesn't have ordinary rain or snow; it rains juice or snows things like mashed potatoes.
20. 曾经有一座宫殿，被一个大花园环绕着。宫殿附近的天气一直都很好，土壤也很肥沃，但花园里却长不出东西。花园里的植物既不开花也不结果，连树上的叶子都很少，也没有地方可以让人乘凉。English translation :There was once a palace surrounded by a large garden. The weather near the palace was always good, and the soil was fertile, but nothing would grow in the garden. The plants in the garden neither flowered nor bore fruit, and even the trees had few leaves, leaving no place for people to find shade.
21. 角落里的那个房子非常吓人，窗户上长满了蜘蛛网，屋顶是歪的，门半开着，轻轻推一下就会发出吱呀的响声。在满月或暴风雨来临时，风穿过灰色墙壁上的洞，会发出低沉的嗡嗡声。English translation :The house in the corner was very scary, with cobwebs covering the windows, a crooked roof, and a half-open door that would creak when pushed slightly. During full moons or approaching storms, the wind passing through the holes in the gray walls would make a low humming sound

22. 阁楼里有一张奇怪的旧地图，上面画了一个在海中央的小岛。从地图可以看到岛上有森林和悬崖。一条长长的河流从黄色的沙滩上流进大海。在河的中段，地图上标着一个大大的记号。English translation :In the attic, there was a strange old map showing a small island in the middle of the sea. From the map, you could see forests and cliffs on the island. A long river flowed from a yellow beach into the sea. In the middle section of the river, the map had a large mark.
23. 学校已经为开学第一天做好了准备。桌子和椅子都已经整齐地摆放在教室里。每张桌子上都整齐地摆放着开学要用的书。但就在开学前一天，洗手间的一根管子破了。整个学校都被水淹没了。English translation :The school was ready for the first day of classes. Tables and chairs were neatly arranged in the classrooms. Each desk had books neatly arranged for the start of school. But the day before school started, a pipe in the bathroom broke. The entire school was flooded with water.
24. 在烹饪学校的窗台上有一个罐子，罐子里放着一团面团。罐子被太阳照了一整天。于是罐子里的面团变得越来越大。很快罐子就装不下面团了，但面团还在不断地变大，慢慢占满了整个窗台。English translation :On the windowsill of the cooking school, there was a jar containing a ball of dough. The jar was in the sun all day. So the dough in the jar grew bigger and bigger. Soon the jar couldn't contain the dough anymore, but the dough kept growing, slowly filling the entire windowsill.

Degraded speech condition (created by the authors)

1. 一天，小丽在森林里发现了一只会说话的松鼠。松鼠告诉她，只要她能在日落前找到一颗金色的橡果，就能实现一个愿望。小丽找遍了整个森林，却只找到了一颗普通的橡果。English translation :One day, Xiao Li discovered a talking squirrel in the forest. The squirrel told her that if she could find a golden acorn before sunset, she would be granted one wish. Xiao Li searched the entire forest but only found an ordinary acorn.

2. 小明的爷爷给了他一块古老的怀表，说这块表能带他去任何他想去的地方。小明半信半疑地转动了表针，突然发现自己站在了火星上。他惊讶地看着周围的红色沙漠，心想这表可能是真的。English translation :Xiao Ming's grandfather gave him an ancient pocket watch, saying this watch could take him anywhere he wanted to go. Xiao Ming skeptically turned the watch's hands and suddenly found himself standing on Mars. He looked around at the red desert in surprise, thinking the watch might be real after all.
3. 小红在阁楼里发现了一本魔法书。她翻开书页，书中的文字突然变成了飞舞的蝴蝶。蝴蝶们围着她飞舞，最后在她手心留下了一颗宝石。小红不知道这颗宝石有什么用，但她决定好好保管它。English translation :Xiao Hong found a magic book in the attic. When she opened the pages, the words suddenly turned into dancing butterflies. The butterflies flew around her and finally left a gem in her palm. Xiao Hong didn't know what the gem was for, but she decided to keep it safe.
4. 一天，小刚在河边钓鱼时，钓到了一只金色的鱼。鱼告诉他，如果他能找到三片不同的叶子，就能获得无尽的财富。小刚立刻开始在河边寻找，但他只找到两片叶子，第三片怎么也找不到。English translation :One day, while Xiao Gang was fishing by the river, he caught a golden fish. The fish told him that if he could find three different leaves, he would gain endless wealth. Xiao Gang immediately began searching along the riverbank, but he only found two leaves and couldn't find the third one no matter how hard he tried.
5. 小芳在花园里种下了一颗奇怪的种子。几天后，种子长出了一棵会发光的树。每当夜晚来临，树上的光芒会照亮整个花园。小芳的朋友们都被这棵树吸引，纷纷来花园里玩耍。English translation :Xiao Fang planted a strange seed in the garden. After a few days, the seed grew into a glowing tree. Every night, the tree's light would illuminate the entire garden. Xiao Fang's friends were all attracted to this tree and came to play in the garden.

6. 小杰在沙漠中迷路了，突然看到远处有一座闪闪发光的城堡。他走近城堡，发现城堡的门会自动打开。城堡里充满了奇异的宝物。小杰在城堡里探险，最后找到了一扇通往回家的门。English translation :Xiao Jie got lost in the desert and suddenly saw a shining castle in the distance. He approached the castle and found that its doors opened automatically. The castle was full of strange treasures. Xiao Jie explored the castle and finally found a door that led back home.
7. 小丽在图书馆里发现了一本无字的书。她翻开书页，突然书中的空白处开始浮现出画面。画面讲述了一个关于冒险和友谊的故事。小丽被故事深深吸引，最后发现书中的主人公竟然是她自己。English translation :Xiao Li discovered a book without words in the library. When she opened the pages, images suddenly began to appear in the blank spaces. The images told a story about adventure and friendship. Xiao Li was deeply captivated by the story and finally discovered that she herself was the protagonist in the book.
8. 小明的爸爸给了他一把神奇的钥匙，说这把钥匙能打开任何一扇门。小明试着用钥匙打开家里的每一扇门，最后发现他能打开通往另一个世界的门，小明决定经常去那里探险。English translation :Xiao Ming's father gave him a magical key, saying this key could open any door. Xiao Ming tried using the key to open every door in his house, and finally discovered he could open a door to another world. Xiao Ming decided to explore that world often.

Children

Intact speech (revised from materials in the adult experiment)

1. 海盗告诉了小明一个宝藏秘密,说他觉得宝藏就埋在小明家。小明相信了他,在家后面挖了个一个大洞,但他没找到宝藏。小明意识到原来海盗也不知道宝藏在哪。English translation :A pirate told Xiao Ming a treasure secret, saying he thought the treasure was buried at Xiao Ming's home. Xiao Ming believed him and dug a big hole behind his house, but he didn't find any treasure. Xiao Ming realized that the pirate didn't know where the treasure was either.

2. 两个女孩在森林里散步。她们看到一个长胡子怪物躺在石头旁。女孩们以为怪物的胡子被石头压住了，便剪掉胡须想救它。 但怪物认为胡子被毁，他非常生气。English translation :Two girls were walking in the forest. They saw a monster with a long beard lying beside a rock. The girls thought the monster's beard was trapped under the rock, so they cut off his beard to save him. But the monster felt his beard was ruined, and he was very angry.
3. 从前，有一个女孩和她的弟弟去采花。天气很热，弟弟非常口渴。在回家的路上，他喝了一个被下过魔咒的水坑里的水，喝完水后，男孩变成了一只小山羊。English translation :Once upon a time, a girl and her little brother went to pick flowers. The weather was hot, and the brother was very thirsty. On their way home, he drank water from a puddle that had been cursed, and after drinking the water, the boy turned into a little goat.
4. 小明的妈妈让他去湖边捉一条鱼当作今天的晚餐。小明害怕一个人去湖边，他觉得湖里有水怪。妈妈告诉小明那只是他自己的倒影，可以试着对水怪笑一笑。English translation :Xiao Ming's mother asked him to go to the lake to catch a fish for dinner. Xiao Ming was afraid to go to the lake alone, thinking there was a water monster in the lake. His mother told Xiao Ming it was just his own reflection and suggested he try smiling at the water monster.

Degraded speech

1. 小丽在森林里发现了一只会说话的松鼠。松鼠告诉她，只要她能在找到一颗金色的橡果，就能实现一个愿望。小丽找遍了整个森林，却只找到了一颗普通的橡果。English translation :Xiao Li discovered a talking squirrel in the forest. The squirrel told her that if she could find a golden acorn, she would be granted one wish. Xiao Li searched the entire forest but only found an ordinary acorn.
2. 爸爸给了小明一把钥匙，说这把钥匙能打开任何门。小明试着用钥匙打开家里的每一扇门，最后发现他能打开通往另一个世界的门，小明决定经常去那里探险。English translation :Dad gave Xiao Ming a key, saying this key could open any door. Xiao Ming tried using the key to open every door in his house, and

finally discovered he could open a door to another world. Xiao Ming decided to explore that world often.

3. 小杰在沙漠中迷路了，突然看到一座闪闪发光的城堡。他走近城堡，发现城堡的门打开了。城堡里充满了宝物。小杰在城堡里探险，最后找到了一扇回家的门。English translation :Xiao Jie got lost in the desert and suddenly saw a shining castle. He approached the castle and found that its doors opened. The castle was full of treasures. Xiao Jie explored the castle and finally found a door home.
4. 小芳在花园里种下了一颗种子。几天后，种子长出了一棵发光的树。每天晚上，树上的光会照亮整个花园。小芳的朋友们都被这棵树吸引，纷纷来花园里玩耍。English translation :Xiao Fang planted a seed in the garden. After a few days, the seed grew into a glowing tree. Every evening, the light from the tree would illuminate the entire garden. Xiao Fang's friends were all attracted to this tree and came to play in the garden.

The distribution of identified channels for each type of fCOI

Figure S1: Child LAN Left

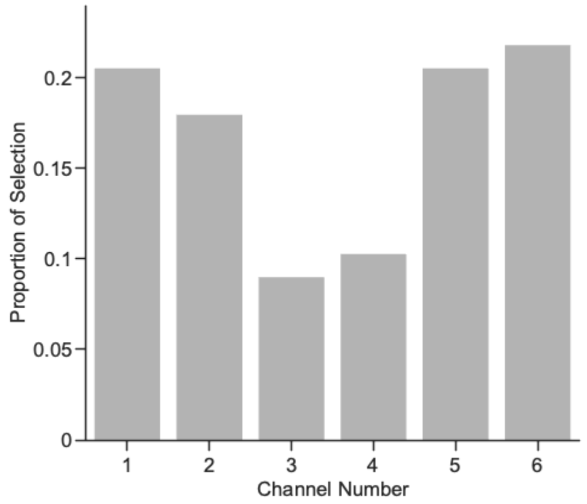

Figure S2: Child LAN Right

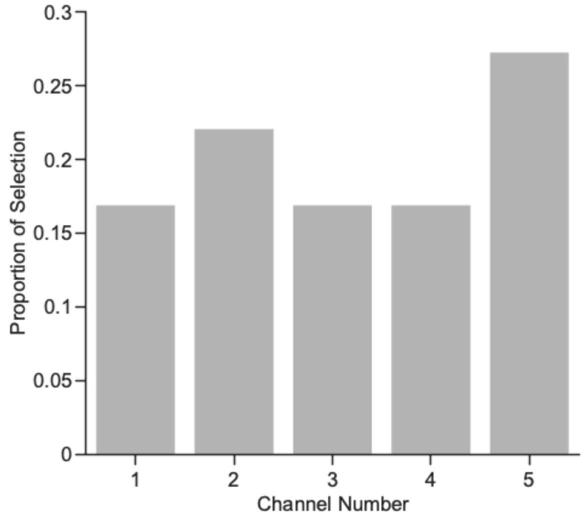

Figure S3: Child MD left

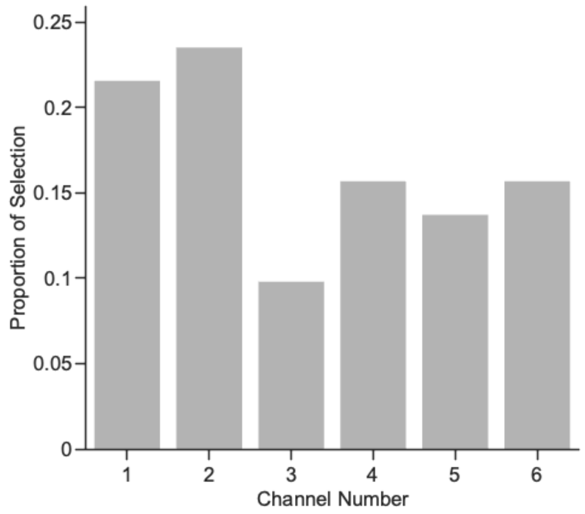

Figure S4: Child MD Right

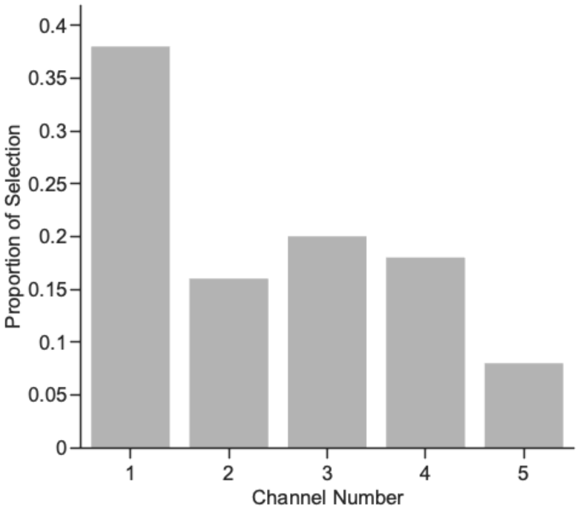

Figure S5: Adult LAN left

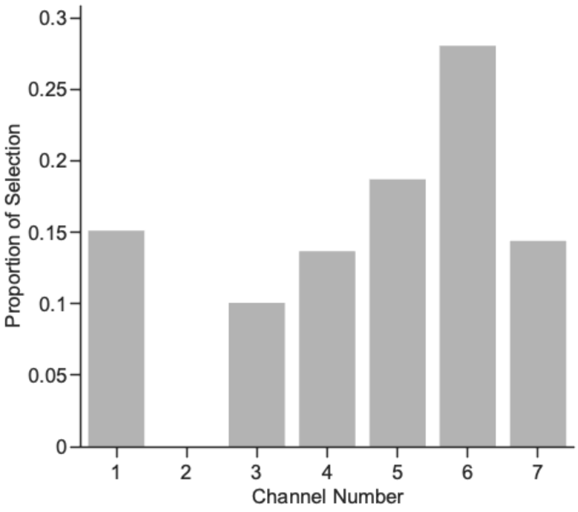

Figure S6: Adult LAN Right

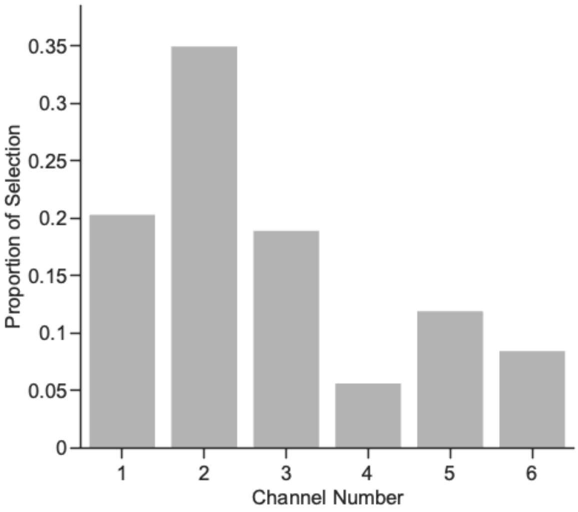

Figure S7: Adult MD left

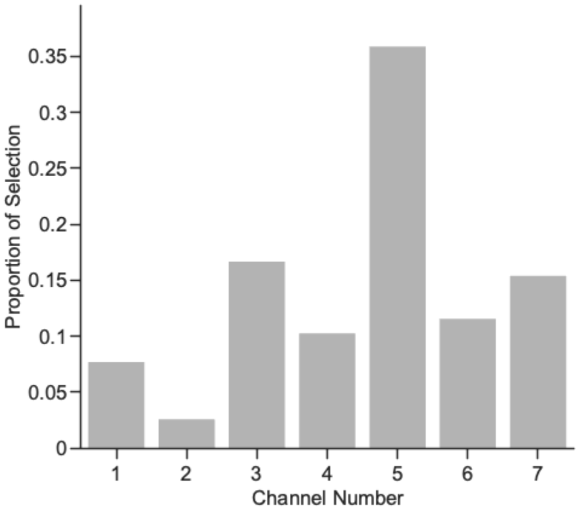

Figure S8: Adult MD right

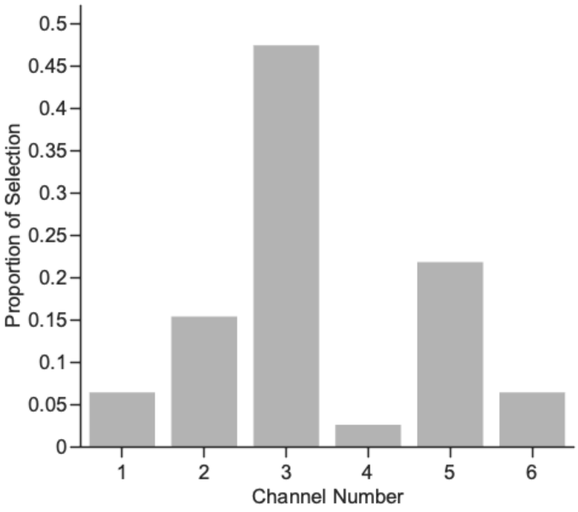

Supplementary Results: Deoxygenated Hemoglobin (HbR) Analyses

To complement our primary analyses of oxygenated hemoglobin (HbO), we also examined the patterns of deoxygenated hemoglobin (HbR) changes. In a canonical hemodynamic response, activation is associated with an increase in HbO and a corresponding decrease in HbR. We report the HbR results for both adult and child participants below, applying a significance threshold of $\alpha = 0.025$.

Adults

After correcting for multiple comparisons, the HbR results in adults did not show any significant effects.

- **Language fCOIs:**
  - The **LIFG language fCOI** showed no significant effect of condition in either the language task (Intact vs. Degraded speech; $\beta = 2.44e-06$, SE = 2.55e-06, $t = 0.96$, $p = 0.345$) or the MD task (Hard vs. Easy WM; $\beta = -1.91e-06$, SE = 5.14e-06, $t = -0.37$, $p = 0.710$).
  - Similarly, the **RIFG language fCOI** showed no significant modulation in the language task ($\beta = -1.37e-06$, SE = 2.45e-06, $t = -0.56$, $p = 0.579$) or the MD task ($\beta = 6.45e-06$, SE = 3.88e-06, $t = 1.66$, $p = 0.098$).
- **MD fCOIs:**

  - The **LIFG MD fCOI** did not show a significant effect for the harder versus easier WM conditions (β = -1.48e-05, SE = 6.34e-06, t = -2.33, p = 0.026). It also showed no significant effect when tested on the language task (β = -3.87e-06, SE = 3.55e-06, t = -1.09, p = 0.277).

  - Similarly, the **RIFG MD fCOI** also showed no significant modulation for the harder WM condition (β = -1.05e-05, SE = 4.65e-06, t = -2.26, p = 0.029) and no significant effect during the language task (β = -2.14e-06, SE = 2.58e-06, t = -0.83, p = 0.407).

Children

The HbR results in toddlers revealed several significant effects, though they did not consistently show the canonical inverse relationship to the HbO findings.

- **Language fCOIs:**

  - The **LIFG language fCOI** did not show a significant effect for either the language task (Intact vs. Degraded speech; β = 6.75e-06, SE = 8.38e-06, t = 0.81, p = 0.425) or the Go/no-go task (Hard vs. Easy; β = -2.24e-06, SE = 6.01e-06, t = -0.37, p = 0.711).

  - The **RIFG language fCOI** also showed no significant effect for the language task (β = 5.48e-06, SE = 1.09e-05, t = 0.50, p = 0.617). However, when tested on the Go/no-go task, this fCOI showed an unexpected significant increase in HbR for the harder condition (β = 2.27e-05, SE = 8.74e-06, t = 2.60, p = 0.011).

- **MD fCOIs:**

  - The **LIFG MD fCOI** showed a significant decrease in HbR during the Go/no-go task (β = -1.25e-05, SE = 4.71e-06, t = -2.66, p = 0.011), mirroring the HbO activation pattern for this condition. It did not show a significant effect for degraded versus intact speech (β = -1.40e-05, SE = 8.94e-06, t = -1.56, p = 0.121).

  - The **RIFG MD fCOI** showed no significant modulation by cognitive demand in the Go/no-go task (β = -1.38e-05, SE = 1.06e-05, t = -1.30, p = 0.202) and no significant effect during the language task (β = 1.19e-05, SE = 9.80e-06, t = 1.22, p = 0.226).

Figure S9. The HbR results in adults did not show significant effects after correction. (a) Left hemisphere language fCOI responses showing no significant difference between speech conditions (p = 0.345) and no significant modulation by cognitive demand (p = 0.710). (b) Right hemisphere language fCOI responses showing no significant difference between speech conditions (p = 0.579) and no significant modulation by cognitive demand (p = 0.098). (c) Left hemisphere MD fCOI responses showing no significant modulation by cognitive demand (p = 0.026) or by speech condition (p = 0.277). (d) Right hemisphere MD fCOI responses showing no significant modulation by cognitive demand (p = 0.029) or by speech condition (p = 0.407). Error bars represent standard error of the mean.

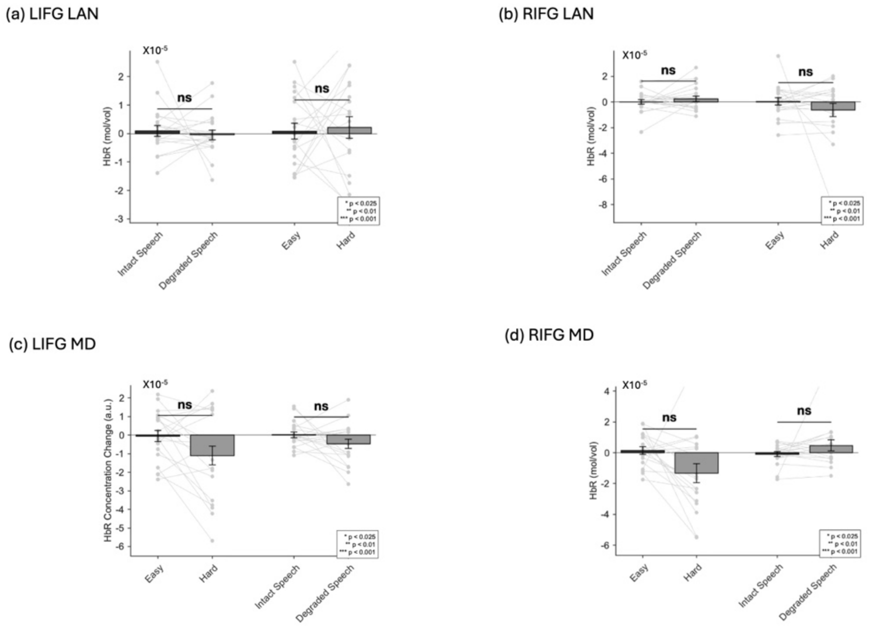

Figure S10. Results from Experiment 2 (Toddlers): Mean HbR responses in the selected fCOIs. (a) Left hemisphere language fCOI responses showing no significant modulation by speech condition (p = 0.425) or cognitive demand (p = 0.711). (b) Right hemisphere language fCOI responses showing no significant difference between speech conditions (p = 0.617) but an significant increase in response to cognitive demand (p = 0.011). (c) Left hemisphere MD fCOI responses showing a significant modulation by cognitive demand (p = 0.011) but no significant difference for degraded compared to intact speech (p = 0.121). (d) Right hemisphere MD fCOI responses showing no significant modulation by cognitive demand (p

= 0.202) or speech condition (p = 0.226). Error bars represent standard error of the mean.

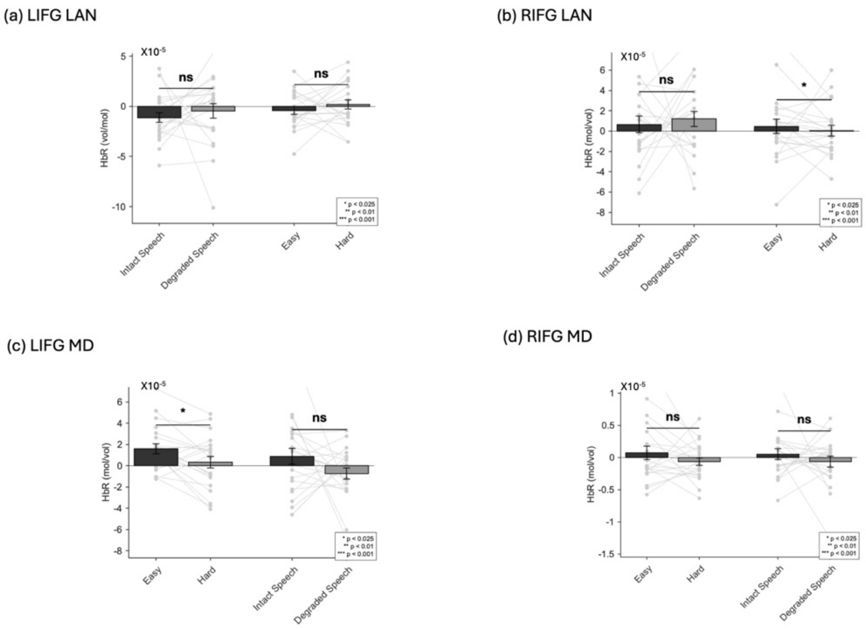

FIGURE S11 Hemodynamic time courses in adult fCOIs (Experiment 1). Grand-average change in HbR concentration (lines) ± SEM (shading) for each fCOI type. Time is in seconds relative to block onset.

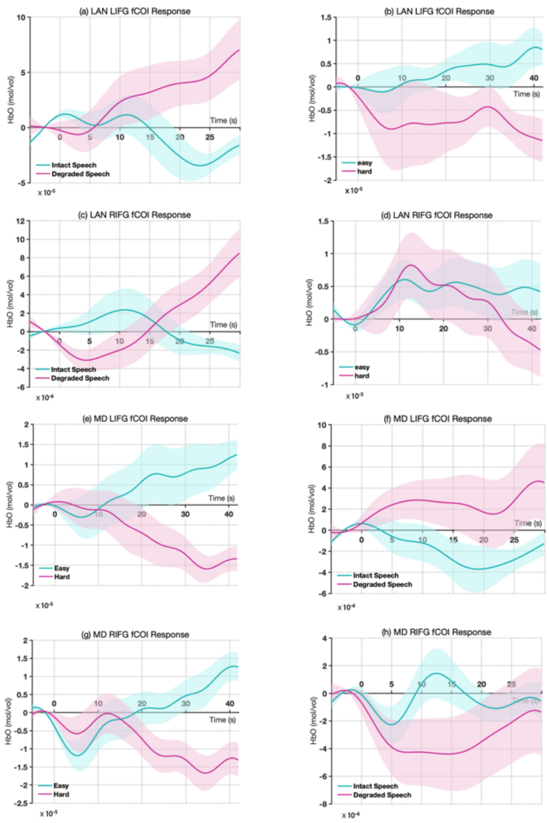

FIGURE S12 Hemodynamic time courses in Toddler fCOIs (Experiment 2). Grand-average change in HbR concentration (lines) ± SEM (shading) for each fCOI type. Time is in seconds relative to block onset.

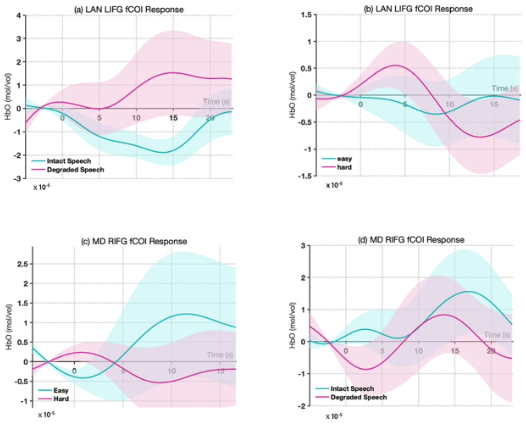

Exploratory analysis: Age-related effects in experiment 2

To further explore potential developmental modulations in our toddler sample, we ran two separate generalized linear mixed-effects models incorporating age as a continuous predictor. For the MD fCOI (right IFG), the model revealed a robust main effect of cognitive condition ($\beta = 4.48 \times 10^{-7}$, $SE = 1.48 \times 10^{-7}$, $p = 0.0032$), while neither the main effect of age ($\beta = 1.23 \times 10^{-11}$, $SE = 4.93 \times 10^{-10}$, $p = 0.980$) nor any age-by-condition interaction was significant. In contrast, the model for the language fCOI (left IFG) showed a marginal main effect of age ($\beta = -1.01 \times 10^{-9}$, $SE = 5.70 \times 10^{-10}$, $p = 0.077$) alongside a significant interaction between condition and age ($\beta = 1.70 \times 10^{-9}$, $SE = 8.06 \times 10^{-10}$, $p = 0.037$). However, subsequent analysis of the simple slopes within each condition did not yield clear evidence of reliable age-related changes. These findings tentatively suggest that although the foundational functional organization in these networks appears to be established by age 2, subtle developmental refinements in language processing may still occur during early childhood. It is important to note that the absence of clear age effects might partly result from our use of fixed preprocessing parameters across all participants. Such an approach may not fully adjust for age-dependent differences in hemodynamic response properties and skull

thickness, potentially masking genuine developmental trends. Future studies employing age-specific analytical strategies may help clarify these subtleties.
